# Alginate as a Promising Biopolymer in Drug Delivery and Wound Healing: A Review of the State-of-the-Art

**DOI:** 10.3390/ijms23169035

**Published:** 2022-08-12

**Authors:** Mohammad A. S. Abourehab, Rahul R. Rajendran, Anshul Singh, Sheersha Pramanik, Prachi Shrivastav, Mohammad Javed Ansari, Ravi Manne, Larissa Souza Amaral, A. Deepak

**Affiliations:** 1 Department of Pharmaceutics, College of Pharmacy, Umm Al Qura University, Makkah 21955, Saudi Arabia; 2 Department of Pharmaceutics and Industrial Pharmacy, Faculty of Pharmacy, Minia University, Minia 11566, Egypt; 3 Department of Mechanical Engineering and Mechanics, Lehigh University, 19 Memorial Drive West, Bethlehem, PA 18015, USA; 4 Department of Chemistry, Baba Mastnath University, Rohtak 124021, Haryana, India; 5 Department of Biotechnology, Bhupat and Jyoti Mehta School of Biosciences, Indian Institute of Technology Madras, Chennai 600036, Tamil Nadu, India; 6 Department of Pharmaceutics, National Institute of Pharmaceutical Education and Research (NIPER), Sector 67, S.A.S. Nagar, Mohali 160062, Punjab, India; 7 Bombay College of Pharmacy, Kolivery Village, Mathuradas Colony, Kalina, Vakola, Santacruz East, Mumbai 400098, Maharashtra, India; 8 Department of Pharmaceutics, College of Pharmacy, Prince Sattam Bin Abdulaziz University, Al-Kharj 11942, Saudi Arabia; 9 Chemtex Environmental Lab, Quality Control and Assurance Department, 3082 25th Street, Port Arthur, TX 77642, USA; 10 Department of Bioengineering, University of São Paulo (USP), Av. Trabalhador São Carlense, 400, São Carlos 13566590, SP, Brazil; 11 Saveetha School of Engineering, Saveetha Institute of Medical and Technical Sciences, Chennai 600128, Tamil Nadu, India

**Keywords:** alginate, drug delivery system, formulations, administration route, controlled release, wound healing, extraction methods

## Abstract

Biopolymeric nanoparticulate systems hold favorable carrier properties for active delivery. The enhancement in the research interest in alginate formulations in biomedical and pharmaceutical research, owing to its biodegradable, biocompatible, and bioadhesive characteristics, reiterates its future use as an efficient drug delivery matrix. Alginates, obtained from natural sources, are the colloidal polysaccharide group, which are water-soluble, non-toxic, and non-irritant. These are linear copolymeric blocks of α-(1→4)-linked l-guluronic acid (G) and β-(1→4)-linked d-mannuronic acid (M) residues. Owing to the monosaccharide sequencing and the enzymatically governed reactions, alginates are well-known as an essential bio-polymer group for multifarious biomedical implementations. Additionally, alginate’s bio-adhesive property makes it significant in the pharmaceutical industry. Alginate has shown immense potential in wound healing and drug delivery applications to date because its gel-forming ability maintains the structural resemblance to the extracellular matrices in tissues and can be altered to perform numerous crucial functions. The initial section of this review will deliver a perception of the extraction source and alginate’s remarkable properties. Furthermore, we have aspired to discuss the current literature on alginate utilization as a biopolymeric carrier for drug delivery through numerous administration routes. Finally, the latest investigations on alginate composite utilization in wound healing are addressed.

## 1. Introduction

In the past, there had been a hurdle in the investigation to reveal naturally derived polymers with exceptional physicochemical characteristics and a high magnitude of compatibility for applications in drug delivery. Escorted by the advancement in pharmaceutical discovery methods in recent years, several therapeutically active substances have come to notice. Nevertheless, the curative agent’s delivery to the intentional site has been a severe hurdle in addressing many ailments. These novel pharmaceuticals need exquisite drug delivery systems (DDSs) that could be employed to improve their pharmacokinetics and pharmacodynamics characteristics, thereby advancing cell/tissue specificity along with their biocompatible properties. Therefore, the blooming of an effective DDS that can transport and administer an active accurately and safely to the desired site of action has to become the “bourne” of scientists.

A drug delivery system (DDS) refers to a system that carries curative substances inside the body to accomplish a required remedial outcome. Two principal classes of DDSs are identified, namely, conventional drug delivery systems and novel drug delivery systems (NDDSs) [1]. The actives are supplied by the conventional method via different routes such as oral, buccal/sublingual, rectal, intravenous, subcutaneous, and intramuscular. The conc. of therapeutic actives is not persistent during the therapy and demands continual dosage management in the conventional route [2]. Hence, this describes the prompt enhancement in the level of the drugs in the blood beyond the toxicity limit after individual administration and later declines to a sub-therapeutic level until the following administration [3]. The enhancement in actives conc. beyond the toxicity limit leads to perniciousness in the body. Moreover, the increase of repeated administration might sum up to the remedial non-compliance upon the sufferer [4].

**Figure 1:**
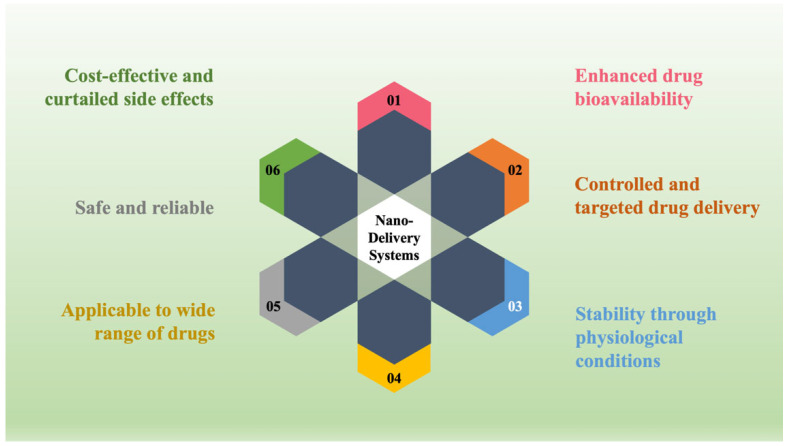
The ideal characteristics of nano delivery systems.

To surmount the aforementioned limitations of the conventional approach, the progressive approach, NDDS, was prepared and included dosage forms. Consequently, the drug rate is sustained within the therapeutic-efficient level with controlled release of actives in both speed and period. Moreover, the NDDS transports actives to the particular action site with optimal dose and diminished toxic effect in contrast to the traditional drug delivery systems [4,5]. The convenient features of the NDDS (as pictured in Figure 1) encompass actives’ controlled release, the capability to utilize different administrative ways, improved active guard and efficiency, the improved substrate solubility showing low solubility, and a novel business market prospective to retrieve pharmaceuticals that have been unsuccessful throughout the traditional drug delivery approaches [1,6,7].

Out of diverse mechanisms of delivery, “controlled drug delivery” and “targeted drug delivery” have been sighted as some of the utmost challenging and fast-progressing investigational areas in the past four years. It provides myriad benefits in contrast to conventional systems, e.g., it improves the absorption rate and biocompatible properties, enhances the actives protection against proteolytic enzyme degradation, cell and tissue-specific active targeting, and helps to regulate active levels within the body, inside the therapeutic level range, over a more prolonged time [8,9]. However, despite the advantages of controlled releases that were formerly persuasive, potential shortcomings, for example, toxicity inside the body, complicated synthetic pathways and the resulting degradation by-products, and operative methods required to explant systems that are non-biodegradable, persist as severe impediments [10,11].

In NDDSs, the active carrier is a base that permits actives to be carried to the intended location, delivering the actives in a controlled manner, thereby enhancing the active bioavailability [12]. Nanoparticles, liposomes, microspheres, polymeric micelles, etc., are some of the significant actives carriers utilized in NDDSs [13,14,15,16,17,18].

Nonetheless, due to their suitable, variable characteristics, polymeric biomaterials are the most alluring opportunity for delivering drugs in a controlled and directed manner. They can be produced on an industrial scale and readily customized to meet the required applications [19]. But the polymer selection utilized for the drug carrier preparation performs a critical function in the process of actives delivery. The two kinds of polymers that are obtainable in the market are natural and synthetic polymers. Natural polymers (for example, chitosan, alginate, and bacterial cellulose), as well as many synthetic polymers such as poly(lactic-co-glycolic acid) (PLGA), poly-L-lysine (PLL), polycaprolactone (PCL), etc., are used as carriers for drug delivery. These polymers have less toxicity, are biocompatible, and are biodegradable, by which they are degraded via the action of enzymes [20,21]. Therefore, natural polymers, such as polysaccharides, polypeptides, or phospholipids, are generally used to prepare a cornerstone. From all of these, alginate (ALG), an anionic polysaccharide, enticed a growing appreciation for actives delivery with its extraordinary physical and biological characteristics. Among different ALGs, sodium alginate (SA) remains one of the most researched in the pharmaceutical area for applications in drug delivery.

## 2. The Purview of the Present Review

This review article comprises state of the art ALG-based preparation in the field of actives delivery. The first segment highlights the exceptional ALG characteristics, trailed by its most recent application in carrying therapeutically active substances. The review acknowledges the research field concentrating on pretty substantial advancement in the past period in medicinal delivery employing ALG and its derivatives as a carrier through various administration routes. Lastly, the review addresses the latest trends in the utilization of ALG composites in wound healing applications.

## 3. Sources of Extraction and Properties of Alginate

**Figure 2:**
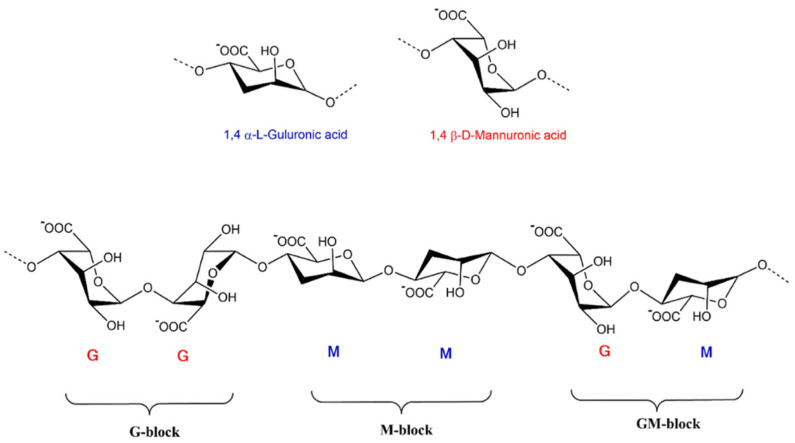
The monomer’s conformation and blocks distribution of ALG salt [27].

ALG, a naturally abundant linear and anionic polysaccharide, is generally obtained from the cell wall of brown seaweed belonging to the class Phaeophyceae [22], including *Ascophyllum nodosum*, *Laminaria hyperborea*, *Laminaria digitata*, *Laminaria japonica*, and *Microcystis pyrifera* [23], and many bacterial strains, including *Acetobacter* and *Pseudomonas* spp. Although it can be created from bacterial origins, it is commercially accessible from algae as SA in its salt form [24]. They are a class of linearly arranged biopolymers comprising 1,4-linked-β-D-mannuronic acid (M-blocks) and 1,4-α-L-guluronic acid (G-blocks) residues ordered in sequences of identical (MM, GG) or heterogeneous (MG) blocks (as portrayed in Figure 2) [25]. Divalent cations, for example, Ba^2+^ and Ca^2+^, can rapidly construct egg-box systems with G block to build ALG hydrogels via the procedure of gelation [26]. Increasing the molecular weight and G-block length dramatically increases the mechanical properties of ALG. Commercially available ALG has an average molecular weight varying between 32,000 and 400,000 g/mol. The ALG solutions have a maximum viscosity at pH 3.0–3.5 because of the hydrogen bonding of the carboxylate groups forming the ALG backbone [23].

**Figure 3:**
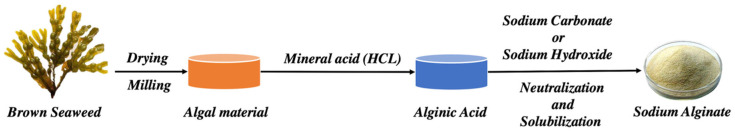
The extraction technique of ALG from brown seaweeds.

Recently, a multistage extraction process (from brown seaweeds, as illustrated in Figure 3) is being carried out, which involves acid pretreatment of the seaweed extract, followed by aqueous alkali treatment (mainly sodium hydroxide) in which different salt forms of natural ALG are modified into aqueous-soluble SA [28]. Subsequently, the filtered extract is incorporated with sodium or calcium chloride, and ALG gets precipitated. Then, dilute HCL is added, and salts of ALG get converted to alginic acid; following further purification and modification, a powder form of water-soluble SA is prepared [29].

The ALG production from bacterial biosynthesis has definite physical characteristics and chemical structures different from the extracted ALG from brown seaweed. The different steps of ALG biosynthesis are (1) synthesis of the precursor substrate, (2) transfer polymerization and cytoplasmic membrane, (3) transport and alteration of the periplasmic membrane, and (4) conveying via the outer membrane [30].

Table 1 denotes the literature survey of various extraction methods of ALG.

ALG’s biocompatibility, rheological properties [40,41], biodegradability, marginal toxicity, and chemical versatility [42] are prominent, along with its exceptional characteristics in producing stable gel in aqueous conditions and a mild environment by adding multivalent cations, making ALG beneficial for drug delivery [43,44]. Furthermore, ALGs can be readily created into various semi-solid or solid frameworks under a moderate environment due to their exceptional sol/gel transition capability. Hence, ALGs are also frequently utilized as viscosity-enhancing substances and thickening agents in the pharma industry [44].

The percentage of the three sorts of blocks—MM, GG, and MG—specifies the ALG’s physical properties. In having a higher percentage of G, ALGs have higher gelling characteristics, while in having a high M content, ALGs have greater viscosity. Determining the M/G ratio is also essential for ALGs, those with a high ratio of M/G produce elastic gels, while those with small M/G ratios produce brittle gels [33,45]. The ALG-based formulation’s mechanical characteristics rigorously rely on the count and conc. of G and M units. If G residues beat M, the formulation exhibits higher mechanical rigidity. Thus, by changing the content of G and M, it is possible to modify the elastic modulus [46].

ALG undergoes hydration at low pH, which leads to the development of “acid gels”, which are highly viscous. The pH sensitivity of ALG can be attributed to acidic pendant groups that accept or release protons due to intermolecular binding when the pH is changed. As a result, the water molecules enter the ALG matrix and get physically entrapped within them but are still free to migrate. This ability is essential in the formation of ALG gels for cell encapsulation [47]. The ALG’s capability to produce two different classes depending on the pH, i.e., acid gel at low pH and ionotropic gel at higher pH, makes it unique compared to neutral molecules [48].

ALG has excellent mucoadhesive characteristics due to the existence of free carboxyl moieties, enabling the biopolymer to attach to mucin via hydrogen bonding as well as electrostatic interaction. On the other hand, ALG solubility is largely dependent on environmental pH and, accordingly, affects their mucoadhesive property since only ionized carboxyl groups are proficient in engaging with tissues of the mucosa. Moreover, soluble ALG assists the penetration of solvent through the polymer matrix, forming a highly viscous and cohesive gel framework for enhancing the mucoadhesive bond strength. In contrast, excessive and exorbitant ALG matrix hydration in physiological solution could diminish mucoadhesive properties due to the weakening of ALG functional groups accessible for mucosal tissue interactions [49,50].

ALGs can be tailored to fulfill the requirements of either pharmaceutical or biomedical applications. Owing to their high water uptake, sustained release, enhanced porosity, and non-immunogenicity, ALGs have found widespread applications in wound dressings [51]. ALG-based composites offer great utility in bioremediation by removing heavy metals, dyes, antibiotics, and other contaminants from wastewater [52]. Based on the types of cross-linkers and the cross-linking approaches used, materials ranging from small drug substances to macromolecular proteins can be designed as controlled drug delivery systems [53].

Biocompatibility is one more vital factor to be studied as the extraction of ALG obtained from nature is accompanied by the existence of numerous impurities capable of inciting allergic responses. In effect, an immune reaction has been stated in industrial-grade ALG; nonetheless, the multi-stage extraction method for eliminating metallic impurities and polyphenolic substances permits the acquisition of ALG of substantially high purity for use in biomedical applications [54].

ALG’s antioxidant and anti-inflammatory actions have also been noticed. It has been reported that ALG oligosaccharides reduce nitric oxide, reactive oxygen species (ROS), and eicosanoids, such as prostaglandin E2 and cyclooxygenase COX-2 production [55,56,57]. Thus, ALG’s exceptional characteristics have unlocked the doorways in its widespread actives delivery applications.

## 4. Hydrogel Formation Methods

In biomedicine, ALG is commonly utilized in the form of a hydrogel for wound healing, medicament delivery, and tissue regeneration applications. Hydrogels refer to highly cross-linked 3D networks comprising hydrophilic polymers. Because hydrogels are physically comparable to biomacromolecular constituents, they are frequently biocompatible and can be administered into the body by non-invasive administration. Hydrogels are commonly formed from hydrophilic polymers by chemical and/or physical cross-linking; their physicochemical characteristics depend on the type of cross-linking, the density of the cross-linker, and the polymers’ chemical composition and molecular weight [58]. We present a review of different methods for cross-linking ALG sequences to generate gels and the effects of these methods on the hydrogel features crucial in the biomedical field.

### 4.1. Ionic Cross-Linking

Combining an aqueous ALG mixture with ionically cross-linking reagents, particularly divalent cations (i.e., Ca^2+^), is the most frequent approach to preparing hydrogels. It is thought that divalent cations attach distinctly to the guluronate block polymers of the ALG chains because the guluronate blocks’ structure permits a good extent of divalent ion coordination. Acknowledged as the egg-box fashion of cross-linking, the guluronate units of one ALG chain form bonds with the guluronate blocks of neighboring polymer chains, culminating in a gel structure [59]. Calcium chloride (CaCl_2_) is a commonly utilized ionic cross-linking agent for ALG. Given the high solubility in aqueous solutions, it often drives fast and poorly regulated gelation. One option is to use a phosphate-rich buffer (e.g., sodium hexametaphosphate) to restrict and control gel formation. These phosphate molecules throughout the buffer compete with the ALG’s carboxylate groups to interact with calcium cations, thus slowing the gelation process [60].

When utilizing divalent cations, the rate of gelation plays an important role in affecting gel strength and consistency; a higher gelation rate results in more homogenous structures and enhanced mechanical character [61]. The temperature at which gelation occurs impacts the gelation speed and the gels’ material performance; the typical reactivity of ionic cross-linkers (e.g., Ca^2+^) is lowered at lower temperatures and consequently hampers the cross-linking. The cross-linked framework that results has more order, which ameliorates mechanical characteristics [46]. Furthermore, determined by the ALGs’ chemical composition, the mechanical properties of ionically cross-linked ALG hydrogels might vary dramatically. For instance, ALG hydrogels made from a large concentration of G blocks are stiffer than those made from ALG with a lower number of G blocks [62].

Ionotropically cross-linked ALG hydrogels incorporating S-nitroso-mercaptosuccinic acid (S-nitroso-MSA), a NO donor, and AgNPs produced from green tea were prepared by Urzedo et al. using glycerol as a plasticizer, which enhanced the flexibility of the hydrogel. Rheological analysis revealed that G′ (elastic modulus) > G″ (viscous modulus) at all temperatures, indicating the prevalence of a solid-like gel structure with thermal stability. Relative to control ALG hydrogel, ALG hydrogels with AgNPs had a modest drop in elastic modulus (G′), leading to a lowering of the elastic response of the resulting nanocomposite gels, manifesting that the addition of AgNPs reinforces the hydrogel network while also preventing the creation of cross-linked junctions between ALG chains. As a consequence, the degree of stress required to break the elastic framework may be reduced. Because the ALG hydrogels were physically in the form of gels, the inclusion of MSA and AgNPs had no substantial effect on their rheological properties [63].

Bruchet and Melman demonstrated a reductive cation exchange mechanism for the synthesis of calcium-cross linked hydrogels from iron (III) ALG hydrogels while still maintaining the shape of the initial hydrogel. Hydrogels prepared by the traditional ionic cross-linking method are heterogeneous and difficult to control. They previously reported the selective oxidation of iron (II) cations, which dissolve homogeneously in SA, to iron (III) cations, triggering the formation of hydrogels; the obtained hydrogel films dissolve on electrochemical/photochemical reduction. Patterned films or coatings can be subsequently prepared from the as-prepared hydrogels [64].

Gattás-Asfura et al. studied various 1-methyl-2-diphenylphosphino-terephthalate (MDT) end groups, polar charged groups, polymer size, and material constitution to alter the physical characteristics of the cross-linkers and increase the capability for covalent stabilization of Alg-N3 beads by Staudinger ligation. The breakdown of cross-linkers after bead production was found to be reduced by branched poly(ethylene glycol) (PEG) polymers, albeit this effect was minimized as polymer miscibility was increased. Due to the elevated miscibility and absence of pre-incubation time for the synthesis of covalently stabilized beads, the results suggested that an ALG-based cross-linker gave the most stable and homogenous beads [65].

In another investigation, Awasthi et al. aimed to make a prolonged-release repaglinide encapsulated double cross-linked ALG-pectin bead matrix utilizing Ca^2+^ ions and the bifunctional alkylating agent epichlorohydrin. The dual cross-linked beads showed higher surface smoothness than single-cross-linked ALG beads owing to the higher cross-linking degree. On the other hand, ALG-pectin beads demonstrated a decrease in mucoadhesive strength due to the inability of solvent penetration owing to the rigid matrix [66].

### 4.2. Covalent Cross-Linking

Covalent cross-linking is being studied extensively to enhance the stability of ALG hydrogels for a variety of biomedical applications, with its carboxylate group being the main site of covalent bonding interactions. The cross-links disintegrate and reorganize in another location, and water is removed from the hydrogel, and the stress applied to it relaxes, resulting in plastic deformation. Although water migration can cause stress relaxation in covalently cross-linked gels, the incapability of dissolving and re-establishing bond formation causes considerable elastic deformity. Covalent cross-linking reagents, on the other hand, could be hazardous, and unreacted compounds may require elimination entirely from gels.

The covalent cross-linking of ALG with variable molecular weight poly(ethylene glycol)-diamines was initially studied with the purpose of generating gels with a diverse extent of mechanical behavior. As the elastic modulus grew steadily with the increase in the crosslinking density or weight fraction of PEG in the gel, it then declined as the molecular weight between cross-links (Mc) became lower than that of the softer PEG [67]. By exploring the use of various types of cross-linking agents and regulating the cross-linking densities, it was later proved that ALG hydrogels’ mechanical characteristics and swelling might be finely controlled. As one may imagine, the chemical constitution of the cross-linking chains has a considerable impact on the swelling ability of the hydrogel. The addition of hydrophilic cross-linking compounds (e.g., PEG) as a supporting macromolecule can rationalize the hydrogel’s loss of hydrophilicity due to the cross-linking reaction [68]. Gao et al. employed dual cross-linked methacrylated ALG hydrogel to overcome ionic cross-linked hydrogels’ tendency to be prone to aqueous decomposition. The covalent cross-links between the methacrylate groups prevented the fracture of dual cross-linked chains of the hydrogels prepared under UV irradiation [69].

Basu et al. designed a nanocomposite DNA-based hydrogel via the establishment of reversible imine bonds that were crosslinked with oxidized alginate (OA). Storage moduli, yield stress, yield strain, and swift restoration following the withdrawal of cyclic stress were all highest in formulations containing OA with a higher oxidation degree. Owing to the reversibility of the covalent imine linkages generated between the aldehyde groups of OA and the amine groups found in the DNA molecules, the hydrogel preparations displayed self-recovering and shear-thinning capabilities. The improved hydrogel was effective in enhancing simvastatin’s prolonged release for more than seven days [70].

In another investigation, Xing et al. reported covalently cross-linked carbohydrate-based ALG/chitosan (CS) hydrogel inserted ALG microspheres encapsulating bovine serum albumin (BSA). The gelation occurred due to the Schiff-base reaction between the amino and aldehyde groups of N-succinyl CS (N-Chi) and OA. Higher content of OAlg in the hydrogels resulted in the creation of stable hydrogels, lowered swelling degree, and higher compression strength [71].

### 4.3. Photo Crosslinking

Photocross-linking is a novel method of in situ gelation that takes advantage of covalent cross-linking. With suitable chemical precursors, photocrosslinking can be done in mild reaction circumstances, even immediately contacting drugs and cells. ALG hydrogels are transparent and flexible when treated with methacrylate and cross-linked with a laser (argon-ion laser, 514 nm) for 30 s with eosin and triethanolamine treatment [72]. Rouillard et al. employed photocross-linking of methacrylate-modified ALG by the photoinitiator VA-086 in order to get high cell viability scaffolds (>85%) [73]. Bonino et al. monitored the reaction kinetics of methacrylate-modified SA hydrogels cross-linked by UV radiation in the presence of a photoinitiator by in-situ dynamic rheology [74].

In a similar study, Jeon et al. created a bioadhesive with adjustable material characteristics, adhesiveness, and biodegradation rate using a dual crosslinked oxidized methacrylated ALG/8-arm PEG amine (OMA/PEG) hydrogel framework. ALG had been chemically modified by reacting aldehydic groups via oxidation with PEG amino groups, and a proportion of the ALG carboxylate groups was further transformed with 2aminoethyl methacrylate (AEMA) using carbodiimide chemistry to enable photocross-linking of the methacrylate by UV light. When cultivated in the presence of human bone marrow-derived mesenchymal stem cells, the functionalized OMA/PEG hydrogels exhibited cytocompatibility. Furthermore, the adhesiveness of the hydrogels proved to be better than that of commercially available fibrin glue, which can be controlled by altering the oxidation level of ALG and assessed on a pig skin model [75].

### 4.4. Click Chemistry Reactions

Most of the strategies for preparing covalently cross-linked ALG hydrogels employ hazardous chemicals and catalysts that impact the biocompatibility of the hydrogels [76]. Hence, in recent times, copper-free “click” chemical procedures are effectively used to make hydrogels based on biopolymers without any need for catalysts or activators, with few side reactions, and are suitable for physiological environments even under mild conditions [77]. The most typical example is 1,3-dipolar cycloadditions, a copper (I)-catalyzed reaction of azides with alkynes, which has recently been used to generate biodegradable peptide-modified ALG hydrogels exhibiting usefulness as artificial analogous to extracellular matrix in tissue regeneration [78].

Anugrah et al. prepared novel near-infrared light-sensitive ALG hydrogels via click cross-linking by inverse electron demand Diels–Alder reaction between norbornene-modified ALGs and tetrazine cross-linkers consisting of diselenide bonds. Indocyanine green (ICG) produced reactive oxygen species upon NIR light irradiation that dissolved diselenide linkages in the hydrogel network, causing the gel-sol transition followed by the release of encapsulated DOX [79]. García-Astrain et al. synthesized cross-linked ALG hydrogels by means of Diels–Alder (DA) click chemistry. Furan groups were added to ALG by an amidation reaction using furfuryl amine. The ALG-containing furan was subsequently cross-linked utilising a DA reaction and the polymer poly(propylene oxide)-b-poly(ethylene oxide)-b-poly(propylene oxide). As a bifunctional crosslinking agent, bismaleimide was used. The hydrogels as-prepared displayed rapid pH responsiveness and pulsatory activity between acidic and alkaline conditions, both of which were important characteristics for drug delivery of the model drug vanillin [80]. Coupling DA click chemistry with the thiol–ene reaction, antibacterial SA hydrogels SA/PEG–HHC10 were developed and manufactured by Wang et al. The cysteinyl-terminated antibacterial polypeptide HHC10–CYS (HHC10) was seeded employing the thiol–ene mechanism between the oxy-norbornene group and the thiol group after the hydrogels were made through DA click chemistry with high mechanical properties. The antibiotic hydrogels had a significant antibacterial effect (sterilization rate after 24 h was almost 100%) and excellent biocompatibility [81]. Lückgen et al. rendered the norbornene-tetrazine click cross-linked ALG hydrogels hydrolytically-degradable by oxidation of the backbone with sodium periodate in order to regulate the rheological, physicomechanical, and degradation properties. The produced hydrogels were suitable for cell seeding in 2D and encapsulating in 3D, as evidenced by cell number constancy and excellent viability preservation [82]. In a different study, Pérez-Madrigal et al. fabricated strong ALG/hyaluronic acid (HA) thio-yne click-hydrogel tissue engineering scaffolds which exhibited remarkable mechanical properties (the maximum compressive strength was 1.4 ± 0.55 MPa, with the strain at the break being around 97%; after 7 days, the hydrogels swelled to 198 ± 5.3%) and cytocompatibility [83].

### 4.5. Thermal Gelling

As a result of their customizable temperature-responsive swelling capabilities, thermo-sensitive hydrogels have recently been intensively explored in various pharmaceutical applications. This allows for pro re nata control of drug delivery from the gels [84]. The most widely used thermo-sensitive gels are poly(N-isopropyl acrylamide) (PNIPAAm) hydrogels, which undergo a reversible phase change in aqueous solutions at body temperature (low critical solution temperature near 32 °C). Notwithstanding the significance of thermo-responsive hydrogels in biomedical pertinence, several ALG-based systems have been documented thus far because ALG is not intrinsically thermo-sensitive. In situ copolymerization of N-isopropyl acrylamide (NIPAAm) with poly(ethylene glycol)-co-poly(-caprolactone) (PEG-co-PCL) macromer by the addition of SA was used to create semi-interpenetrating polymer network (semi-IPN) framework. At a fixed temperature, the swelling capacity of the gels rose with the amount of SA and reduced with a temperature rise. The introduction of SA in semi-IPN structures enhanced the mechanical properties and BSA release from the hydrogels, suggesting that it could be advantageous in drug delivery [85]. Bezerra et al. prepared furosemide-loaded sericin/ALG beads via ionic gelation and then subjected them to thermal or covalent cross-linking using proanthocyanidin as the cross-linker to achieve gastro-resistant sustained release diuretic particles [86].

### 4.6. Cell Cross-Linking

Whereas a variety of chemical as well as physical ways of forming ALG gels have been documented, the potential of cells to promote gel production has largely been overlooked. Despite the lack of chemical cross-linkers, the ability of cells to attach multiple polymer chains can result in longer, reversible network development when ALG is altered with cell adhesion ligands. Cells introduced into an arginine–glycine–aspartic acid (Arg–Gly–Asp, RGD) functionalized ALG solution produce a uniform dispersion, and this system then forms the cross-linked networks without the use of any extra cross-linking chemicals through specialized receptor–ligand associations [23].

Yu et al. produced peptide-modified ALG microspheres encapsulating human mesenchymal stem cells for delivery into injured myocardium. Cell–ECM interface dynamics have an influence on both cell-matrix adhesion but also cellular processes, including migration, growth, maturation, and cytokine and growth factor signaling. In vitro data reveal that hMSCs adhere to the RGD-functionalized ALG surfaces more firmly than the un-modified ALG. Furthermore, compared to the non-modified cohort, the FGF2 expression level on the RGD-treated surface was considerably higher [87].

Similarly, in a study by Fonseca et al., ALG was modified by partial cross-linking with a matrix metalloproteinase cleavable peptide (proline–valine–glycine–leucine–isoleucine–glycine) by carbodiimide chemistry and co-incorporated into cell-adhesive RGD-ALG hydrogels. Matrix metalloproteinase-2 (MMP-2) function was enhanced in MSC grown in ALG functionalized with MMP-sensitive polypeptide; thus, this approach increased their role as ECM analogs in a more flexible and physiological 3-D cell milieu [88].

Other novel methods for ALG gelation include cryogelation, where growing crystals form interconnecting pores, generating solid materials and expelling swelling agents from gels via freeze-drying [89].

In the non-solvent induced phase separation technique, when the solubility of polymers decreases caused by the presence of a non-solvent, a polymeric solution segregates into polymer-heavy and polymer-lean phases. As an outcome, a lyogel is formed, which can be “hardened” by removing the parent solvent further [90,91].

At normal room temperature, a suspension of metal carbonate or hydroxycarbonate (Ca, Sr, Ba, Zn, Cu, Ni, or Co) is exposed to pressured carbon dioxide (30–50 bar) in a process called carbon dioxide-induced gelation. One of the driving forces behind the establishment of CO_2_-induced gelation is to eliminate some of the processes in aerogel manufacture as much as is feasible by merging gelation, solvent exchange, and SC-drying into a single procedure [92].

Another upcoming process of ALG gelation is the so-called carboxylic acid-induced gelation. Gelation was shown to be quick with oxalic, citric, and maleic acids, allowing the formation of gel beads by expelling ALG solution (4% *w*/*v*) into the relevant acidic solution (0.5 M). These findings demonstrate that the acid and ALG chains have such a strong interaction that gel breakdown was not observed after several washes of carboxylic acid-induced crosslinked gels in an aqueous medium at pH 7 [93,94].

Aerogels are an upcoming class of carriers for therapeutic drug delivery, having low density (0.005–0.50 g/cm^3^), high specific surface area (300–1000 m^2^/g), and high porosity (upto 99%) [95,96,97]. Polymers such as starch, cellulose, pectin, ALG, etc., can be used to produce highly adsorption-efficient aerogel microparticles. Lovskaya and Menshutina prepared ALG-based microparticles loaded with three model drugs (loratadine, nimesulide, and ketoprofen) using the supercritical adsorption process. The release rate of drugs in the ALG-based aerogels was found to increase compared to pure drugs [98]. Similarly, Athamneh et al. used aerogel-based microspheres prepared by ALG and ALG-HA hybrids by the emulsion gelation method as carriers for pulmonary drug delivery. These ALG-based microspheres showed an in vitro aerodynamic diameter of 5 µm, indicating the favorable properties of ALG-based microspheres for pulmonary delivery [99,100].

## 5. ALG Particles Formation Methods

ALG gelation occurs via one of two mechanisms: (1) external gelation, in which cations enter the ALG system from the outside, or (2) internal gelation, in which cations escape the ALG structure. ALG-based particles of a variety of dimensions can be produced and categorized into various categories: (1) macroparticles, such as that of drug or vitamin tablets, may be seen by the naked eye; (2) microparticles vary significantly in size from a few microns to several millimeters; and (3) nanoparticles have a diameter ranging between 1 to 100 nm [101,102,103,104]. The most popular method for producing ALG particles is the drop-by-drop expulsion of an ALG solution through a needle in a cationic bath. External gelation is used to create ALG particles with diameters between 500–5000 µm [105,106,107]. The ALG particle size can be lowered, and particles having a smaller size distribution could be generated using several ways depending on this external gelation process, including coaxial laminar airflow at the nozzle, electrostatic fields, and vibrating nozzles [107,108,109,110]. In their study, Patel et al. described a method detailing the impact of ionotropic gelation residence time (IGRT) on the extent of cross-linking of ALG particles with Ca^2+^ ions. They found that to prevent the dissolution of the drug into the bulk cross-linking solution, the IGRT must be kept short which also enhances drug loading [111].

An emulsification–gelation process can also be used to make ALG spheres. Gelled spheres are made by emulsifying an ALG solution in an organic phase, followed by the gelation of the ALG emulsion droplets. External gelating of the ALG emulsion droplets leads to emulsion cracking; however, the spheres aggregate and, as a result, numerous emulsion droplets (W/O/W) are formed [112,113]. Membrane emulsification may yield ALG particles with a quite small size distribution; however, particle production is confined to the micro range (>1 µm), and clogging of the membrane layer, as well as the feasibility of the process scale-up, remains a concern. Mechanical emulsification is the most practical for industry, as it produces ALG particles of all sizes. Nevertheless, the size variation of the ALG particles produced is relatively wide. ALG nanoparticles can be made in various ways, although the majority of them rely on complexation, either through interactions with ions like calcium or negatively charged polyelectrolytes like CS [114] or poly-L-lysine [115]. Complexation is used in other processes as well, such as emulsification, to strengthen nanoparticles. In most cases, complexation will be required as a supplement to obtain the particles. The incorporation of calcium chloride into the ALG solution produces nanoaggregates; generally, the drug is typically added before this step. The size of nanoparticles varies greatly depending on a variety of factors, such as the manufacturing process, preparation parameters, crosslinking reagent, ALG molecular mass, and so on. Because ALG is a negatively charged polyelectrolyte, nanoparticles can range in size from several tens to nanometers, and the zeta potential is generally negative [116].

## 6. Current Advancements in ALG Formulations in Drug Delivery

**Figure 4:**
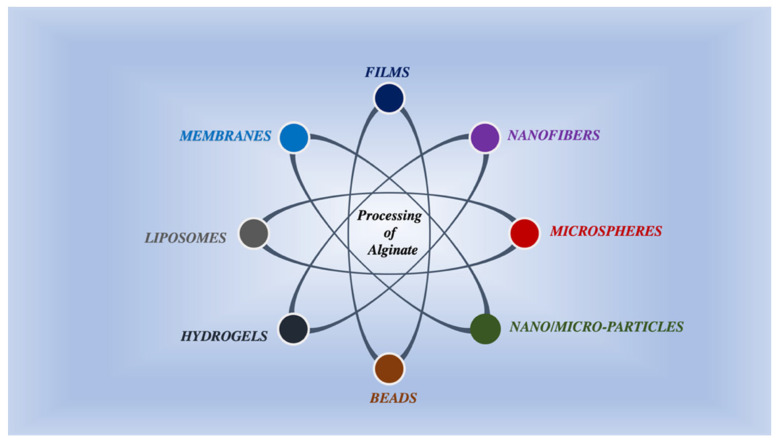
Diagrammatic representation of ALG formulations into diverse forms.

A substantial number of therapeutic actives have been unveiled over the past few years. Numerous of them grasp magnificent therapeutic properties; nevertheless, they manifest poor bioavailability and poor pharmacokinetics, causing detrimental systemic impact after the administration [117]. Therefore, DDS has evolved into an essential device to enhance remedial effectiveness by employing delivery carriers (as illustrated in Figure 4) such as microspheres, hydrogels, beads, liposomes, nanoparticles, nanofibers, etc., to deliver drugs [118,119,120].

**Figure 5:**
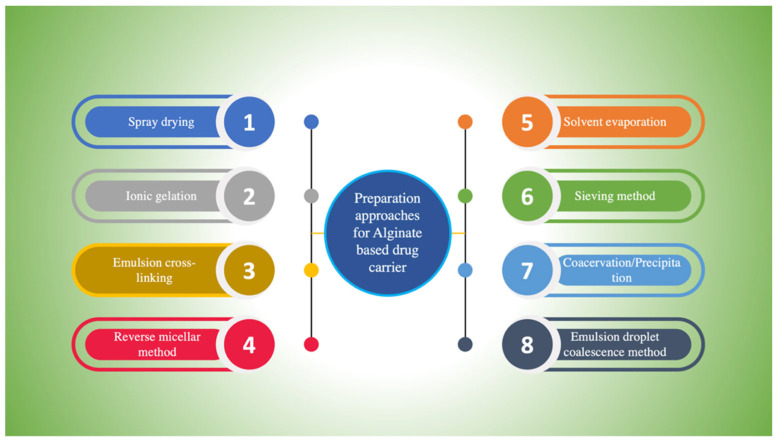
Various approaches for preparing ALG-based particulate carrier matrix.

Different approaches (as shown in Figure 5) are employed to develop the aforementioned drug delivery devices.

Below are a few vital employments of ALG-mediated systems for enhanced actives delivery through various administration routes.

### 6.1. Oral Drug Delivery

Oral drug delivery is the first choice route of drug delivery for any therapeutic substance due to its simplicity, patient compliance, cost-effectiveness, easy bulk manufacturing, sustained and controlled release, and generation of immune response in case of vaccination [121,122,123]. However, some limitations exist, such as variable pH of the gastrointestinal tract (the stomach is highly acidic while the small intestine is alkaline), first-pass metabolism in the liver, the occurrence of hydrolytic enzymes, and an absorption barrier in the liver intestine. This limits the extent of the therapeutic action of many drugs given by the oral route. Nevertheless, due to their non-toxicity, biocompatibility, and biodegradability, ALG hydrogels can be safely administered orally [124].

Recently, Ilgin et al. used SA hydrogels as the carrier for diclofenac sodium to obtain controlled drug delivery at a specific pH. The porous structure made them valuable as a drug delivery system. The researchers prepared SA pH-responsive semi-interpenetrating hydrogels by altering the physical and biological properties of the biocompatible and biodegradable ALG via surface functionalization with the aid of monomers such as HEMA, MAPTAC, and MA. Polyvinyl alcohol (PVA) was employed to provide chemical stability, and nMBA was the copolymer. The resultant hydrogels were evaluated for their swelling, drug loading, drug release, and antimicrobial activity. PVA and HEMA proved to be good polymeric systems because they enhanced biocompatibility and mechanical properties. Maximum swelling (38 g_water_/g_gel_), drug loading (22.8%), and release profiles were observed at pH 7.0, where 95% release of drug was observed at the end of 2 h, as compared to 4.5% at pH 1.5. Antibacterial activity was observed against *E. coli*, *B. subtilis*, and *S. aureus* by the disk diffusion method, and it displayed excellent inhibition of the growth of all three microorganisms. Thus, ALG is confirmed to be a great, intelligent drug release platform for oral drug delivery, providing a controlled release [125].

**Figure 6:**
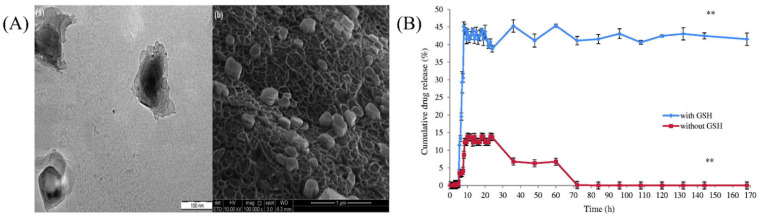

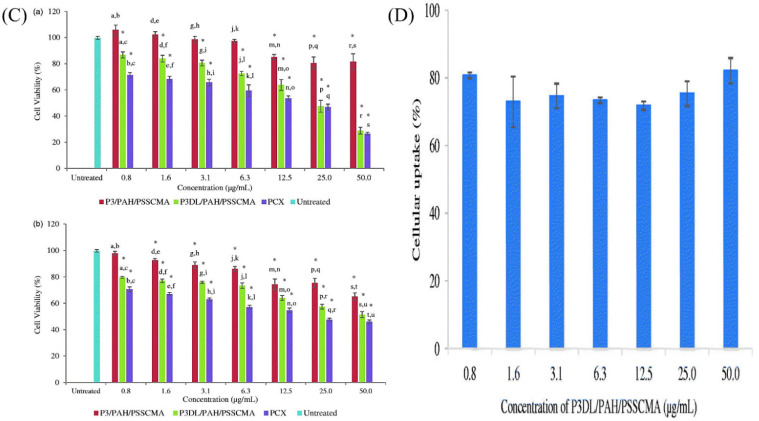
Biocompatible disulfide cross-linked SA derivative nanoparticles for oral colon-targeted drug delivery (**A**) P3DL/PAH/PSSCMA (a) TEM and (b) FeSEM pictures at magnifications of 110,000 and 100,000, respectively. (**B**) For 170 h in a simulated gastrointestinal medium, the cumulative % drug release of P3DL/PAH/PSSCMA was measured. ** means *p* < 0.01. (**C**) The effects of P3/PAH/PSSCMA, P3DL/PAH/PSSCMA, PCX and untreated P3/PAH/PSSCMA on (a) HT-29 and (b) CRL 1790. Data marked with the same letters show significant difference between the samples. * Indicates *p* < 0 .05 compared to the untreated samples. (**D**) P3DL/PAH/PSSCMA nanospheres tagged with rhodamine 110 are taken up by HT-29 cells. Reproduced with permission from [126], copyright Taylor & Francis Online, 2019.

To obtain colon-specific drug delivery for inflammatory bowel diseases such as Crohn’s disease, it is challenging to move through the different pH conditions of the GI tract undisturbed. Hence, Ayub et al. tried to enhance the delivery of Paclitaxel to colonic cancer cells by formulating a self-assembled cysteamine-based disulfide cross-linked biodegradable thiolated SA-derived nanoparticles via a layer-by-layer assembly approach (as shown in Figure 6A). SA was oxidized using sodium periodate before adding cysteamine hydrochloride to alter its backbone. The disulfide bonds prevented the leakage of the drug before it reached its target site. The encapsulation efficiency of P3DL/PAH/PSCCMA was found to be 77.1%, with a cumulative-drug release of 45.1% after 24 h (as displayed in Figure 6B). The nanospheres demonstrated their maximum size at pH 7.0, thus signifying their efficacy in selectively delivering the actives to the colon. The MTT assay revealed the high viability (86.7%) of HT-29 cells (as shown in Figure 6C). More than 70% of the nanospheres were detected in human colon cancer adenocarcinoma HT-29 cells, indicating their high cellular uptake, as displayed in Figure 6D). Stability studies indicated that most nanospheres changed slightly in size and PDI but were stable with negligible differences in zeta potential. Thus, this approach could be employed for colon-targeted drug delivery with minimal toxic effects [126].

In order to furnish controlled drug delivery via the oral route, Cong et al. prepared an ALG hydrogel/CS micelle composite system encapsulating emodin. The research group prepared cross-linked micelles because, unlike conventional polymeric micelles, cross-linked micelles prevent drug leakage and protect them from dissociating quickly. Emodin was chosen as a model drug because it has poor water solubility and undergoes extensive first-pass metabolism. It was loaded onto CS micelles and then mixed with SA hydrogels developed by cross-linking with Ca^2+^ ions and β-GP to form hydrogel/micelle beads. Based on response surface methodology, the optimized biopolymer concentrations were determined. The 1:1 hydrogel/micelle beads showed sustained drug delivery, while the 3:1 ratio provided colon-specific delivery. The morphological, swelling, and degradation studies were carried out. The diameter of micelles increased from 80 nm in aqueous solution to 100–200 nm in the hydrogel, likely due to electrostatic interaction between the amino group of the CS chain and the carboxylate group of the ALG chain. The swelling of micelles was reduced at pH 1.2 due to SA hydrogel, whose swelling ratio is at acidic pH, thus preventing the release and degradation of CS chains. The initial release amount was 28% in SGF, and a final release amount of 85% for 1:1 micelle/hydrogel systems was reported. Thus, this pH-responsive hydrogel/micelle system could be an encouraging candidate for sustained-release or site-targeted actives delivery for unstable or hydrophobic actives [127].

Amphotericin-B is the first-choice drug for many fungal infections, including leishmaniasis. However, it has limited solubility, is susceptible to gastric pH, and its oral bioavailability is low; it is generally administered intravenously [128]. Hence, to overcome this issue, Senna et al. utilized nanostructured lipid carriers (NLC) combined with stimuli-sensitive ALG polymers using a high-pressure homogenization technique with calcium chloride as the cross-linker, providing a dual-benefit of oral drug delivery and protection from gastric pH without structural degradation and promoting drug release at intestinal pH. Nanostructured lipid carriers are systems in which hydrophilic drugs are dispersed in polymeric matrices, forming nanometric droplets, thus enhancing adsorption and preventing enzymatic degradation [129]. AmpB was solubilized by solid lipid glyceryl monostearate (GSM). Studies using the trypan blue exclusion test showed that the NLCs showed low cytotoxicity on Vero cells (ATCC^®^ no. CCL-81™), very high specificity, and their drug release profiles were equivalent to ALG swelling degree profiles, indicating that the drug delivery was primarily due to the polymer’s swelling rate. In the responsive pH range, the carboxylic acid groups of ALGs became ionized and acquired a negative charge, resulting in electrostatic repulsion and allowing water molecules to enter. The NLC particles maintained their framework even after rehydration; thus, the aforementioned system proved promising for the delivery of AmpB orally [130].

Due to its simplicity, non-invasiveness, patient compliance, and economy, oral drug delivery is the most favored route. Particles having a size between 20–100 nm are readily absorbed from the cells while also avoiding renal clearance [131], while particles below 5 nm are quickly cleared via renal clearance. Thus, Thomas et al. prepared ALG-cellulose nanocrystal hybrid nanoparticles by a green method to achieve controlled-drug delivery of the drug rifampicin, an anti-tubercular agent with poor water solubility, which necessitates the search for alternate drug delivery routes. Cellulose nanocrystals (CNCs) were used to improve the mechanical stability, durability, and high diffusion rates due to ALG’s highly porous structure. CNCs have been reported to reduce the voids in the gelatin structure [132]. In addition, CNCs have been shown to enhance the ALG bead’s mechanical strength and improve drug release [133]. Ionotropic gelation was utilized to synthesize ALG-CNC NPs using water as the only solvent. The optimum nanoparticle preparation was found to be with 1% surfactant and a 1:6 ratio of ALG CNC. The drug entrapment efficiency (EE) was between 43–69%. The ALG-CNC-NPs swelled rapidly at pH 6.8 to 7.4. It showed 15% drug release in 2 h at pH 1.2, while at pH 7.4, almost 100% of the drug was released in 12 h, demonstrating its controlled-release profile. It has a negative zeta potential (−15 to −20 mV) due to free carboxylic groups, thus facilitating penetration into epithelial cells. CNCs and ALG-CNC NP’s cytotoxicity was investigated via MTT assay using the L929 fibroblast. It showed 100% cell viability, thus suggesting that these nanoparticles could be a good candidate for drug delivery [134].

The researchers have prepared many formulations and systems to promote actives delivery efficiency via oral routes to beat oral drug delivery limitations. The recent literature includes preparations such as cationic cyclodextrin/ALG/CS nanoflowers/5-fluorouracil [135], hydroxyethylacryl CS/SA hydrogel/paracetamol [136], pH-sensitive nanocomposite using SA/pectin/tannic acid(TA)/silver(Ag)/propranolol [137], Cyperus esculentus starch-ALG/ibuprofen [138], calcium alginate (CA)/SWCNT-GI/curcumin [139], thiol-modified SA microspheres/bovine serum albumin [140], vitamin B12 modified amphiphilic SA nanoparticles/insulin [141], thiolated SA nanoparticles/docetaxel [142], CA beads/cetuximab/octreotide [143], ALG/barium ion/methotrexate [144], CS/CA/liraglutide [145], nano pol cellulose (CMC)/ALG/CS [146].

### 6.2. Ocular Drug Delivery

Topical instillation in the eye is the most preferred non-invasive administration route of actives for various anterior and posterior segment diseases, for instance, glaucoma, uveitis, cataract, and age-related macular degeneration. It is best-loved due to its comfort in administration and being patient-friendly. Nevertheless, the eye has several protective anatomical barriers that limit drug absorption through this route. The lacrimal fluid drains the drug quickly from the ocular area. Hence, ocular drug delivery has the challenge of maintaining the conc. of the drug at the site of action for the necessary time [147,148]. To enhance the delivery of topical ocular therapeutics, researchers have principally highlighted two methods: (i) to improve the period of residence of the cornea by employing viscosity enhancers, mucoadhesive, particulate, and/or in situ gelling systems; and (ii) to enhance the permeability of the cornea using (a) penetration enhancers, (b) prodrugs, and (c) colloidal systems (like NPs and liposomes) [149].

There is a probability of permanent visual damage or blindness due to retinal diseases such as macular degeneration, uveitis, macular edema, etc., and hence, in such cases, immunosuppressant drugs must be provided on a long-term basis to sustain the functioning of the eye. Encapsulated-cell therapy, however, provides a modern approach with long-lasting delivery of newly synthesized protein-rich drugs, eliminating the need for surgical treatment and is potentially removable by surgery. Some limitations pertaining to ECT use are its poor mechanical strength, inadequate biocompatibility, and lack of termination mechanisms [150]. Hence, Wong et al. synthesized an injectable ALG collagen (CAC) hydrogel with an inducible termination switch as the encapsulation matrix for glial-derived neurotrophic factor for safer ocular delivery. Here, collagen acts to enhance the cell viability of ALG. The CAC ECT gels were developed by ionotropic gelation employing calcium chloride, where they used a Tet-on-pro-Casp8 switch mechanism for the effective termination of ECT systems. An oral DOX treatment was sufficient for the termination of the gel system. The therapeutic delivery was evaluated in pink-eyed dystrophic RCS/lav rats, a recessive RP model characterized by a progressive loss of photoreceptors and electrophysiological response. The retinal function of rats acquiring GDNF-secreting gels was found to be better than that of the control group. The ECT gel system was mechanically stable, viable, and functional in vivo. Stability studies revealed that following six months of implantation, the system was mechanically stable, strong, and assisted the growth of various types of cells, viz., HEK293 and ARPE-18 cells. Thus, this approach may be utilized to address a range of posterior eye disorders [151].

Nepafenac is a non-steroidal anti-inflammatory agent utilized to address post-surgery pain in the cornea, such as cataracts. Because of its water insolubility, it is only formulated as a suspension, which leads to irritation in the eye, severe lacrimation, and consequently decreased drug’s residence time due to rapid drainage into the systemic circulation, limiting the conc. of the drug at the action site [152]. Hence, Shelley et al. utilized the ion-activated in-situ gel formation of ALG with Ca^2+^ ions in the lacrimal fluid to accomplish a sustained release of the actives. HPBCD was the actives solubilizer and permeation enhancer, and the retention time and permeation parameters were observed in the ex-vivo porcine perfusion eye model. The in-situ gel formulation showed a considerably increased diffusion rate and permeation rate than the placebo, with a sustained release of over 24 h with release kinetics following the Korsmeyer-Peppas equation [153].

In another investigation, Nagarwal et al. prepared CS-coated SA-CS nanoparticles for the intra-ocular delivery of 5-FU used for corneal carcinoma. It demonstrated an encapsulation efficiency (almost 27%) and drug loading capacity (around 19%). Moreover, the in-vitro and in-vivo drug-release profiles showed a sustained release (8 h) in contrast to the 5-FU solution and also showed good tolerability when tested by the Draize test on the rabbit eye [154].

Conventional ocular drug dosage forms, for example, solutions, suspensions, and ointments, possess certain disadvantages that cause poor absorption of the drug, such as the sweeping activity of eyelids, tears washing away the drug, impermeable endothelium, and blood barrier. In-situ gelling systems are an effective drug delivery and absorption method since the less viscous solution undergoes gelling in the presence of stimuli such as pH, ionic strength, and temperature. Hence, Noreen et al. developed ALG in situ gelling systems using gum obtained from Terminalia arjuna bark with moxifloxacin HCl as the drug for ophthalmic delivery. It undergoes a sol to gel transition at the pH of tear fluid. The preservative was methylparaben, and the osmolarity was adjusted with sodium chloride. The drug was stable and provided sustained release for 12 h. The ex vivo transcorneal penetration was found to be 4.76 ± 0.27%, and corneal hydration was 78.85 ± 0.19%. No ocular irritation was observed [155]. Thus, the overall results demonstrated the aforementioned system as a promising candidate for ocular drug delivery.

In another investigation, Polat et al. formulated nanofibrous ocular inserts for the therapy of bacterial keratitis incorporating the drug Besifloxacin HCl or BH-hydroxypropyl-beta-cyclodextrin (HP-β-CD) complex comprising PCL/PEG fibrous inserts coated with mucoadhesive polymers such as SA or thiolated sodium alginate (TSA) based on the electrospinning method. The coating with SA and TSA increased the insert’s bioadhesion. The preparation demonstrated an initial burst release followed by a slow release for two days, and the thickness and diameter of the inserts were comparable to commercial formulations. Drug loading efficiency was over 90%. Even after seven days of incubation, the inserts did not attain acidic pH values, indicating that the inserts did not cause eye irritation. In vitro studies on ARPE-19 cells on exposure to the ocular inserts demonstrated no cytotoxicity, and their antibacterial activity was comparable to that of commercial formulations. Ex vivo transport research showed that HP-β-CD enhanced solubility and corneal permeability, and the actives delivery was equivalent to commercial formulations [156]. Hence, the overall results reported that the newly prepared system was suitable for ocular drug delivery.

Different researchers have studied various ALG-based preparations for enhanced ocular delivery of drugs. For example, CS/ALG multilayers/diclofenac [157], CS/ALG/daptomycin [158], CS/ALG/acetamiprid [159], ALG/CS/levofloxacin [160], SA/glycerin/flurbiprofen [161], ALG/peppermint phenolic extract [162], SA/methyl cellulose/sparfloxacin [163], ALG/calcium gluconate(CaG)/tryptophan [164], SA dialdehyde/carboxymethyl CS/limbal stem cells (LSC) [165], SA/methyl cellulose/CMC/carbopol/pilocarpine [166], CS/SA/azelastine [167], SA/butyl methacrylate/lauryl methacrylate/linezolid [168] are few of the current investigations which portrayed excellent ocular actives delivery.

### 6.3. Pulmonary Drug Delivery

The lungs are an appealing site for the pulmonary administration of actives via diverse DDSs [169,170]. Moreover, the pulmonary route provides numerous benefits over traditional per oral administration, for instance, greater surface area with fast absorption owing to the high vascularization and evasion of the first-pass metabolism [171]. This selectivity enables targeted actives delivery and, therefore, diminishes the side effects [169]. Nevertheless, the pulmonary route is challenging to deliver the actives deep to the alveolar regions of the lung owing to diverse respiratory obstacles, including mechanical, chemical, pathological, and immunological hindrance [172]. The mechanical barrier employs mucociliary clearance to remove particles (having a mean diameter of >6 µm) [173]. When clearance takes place quicker than absorption, in particular of poorly soluble drugs, the availability of actives in the lungs might be restricted. Additionally, under pathologic states like asthma and COPD, excessive mucus is accumulated in the lung, which inhibits the deeper penetration of actives [173,174]. Furthermore, the chemical barrier containing proteolytic enzymes can degrade the breathed materials, which leads to the destruction of functions of actives and/or delivery carriers. Ultimately, the immunological barrier comprising primarily alveolar macrophages assembles the immunological reactions in the intense lung that eradicate all foreign materials with no difference between potential detrimental materials and advantageous ones. Thus, pulmonary bioavailability and systemic bioavailability for actives provided by maximum traditional pulmonary products are less. Therefore, the progress of unique, more effective DDSs is pivotal to alleviating the consequences of these obstacles [175].

The FDA-approved pulmonary dry powder inhalers are predominantly fast-releasing. To obtain sustained delivery to the lungs, Athamneh et al. developed an aerogel microsphere formulation for suitable delivery to the lower pulmonary tract using the ALG-HA hybrid system by the emulsion gelation method followed by drying with supercritical CO_2_. Combining these approaches resulted in highly porous Alg-HA microspheres with very low density and a high BET-specific surface area. The addition of HA to Alg successfully reduced the particle agglomeration and enhanced its biodegradation, possibly due to the formation of a hydrogen bond between ALG’s carboxylate groups and N-acetyl-glucosamine amide in the hybrid aerogel. In addition, HA was employed to improve the physical characteristics of ALG, such as gelling efficiency and lung tolerance. This led to achieving the desired particle size for pulmonary deposition and improving biodegradability. However, more research using active pharmaceutical ingredients needs to be carried out in this area [100].

**Figure 7:**
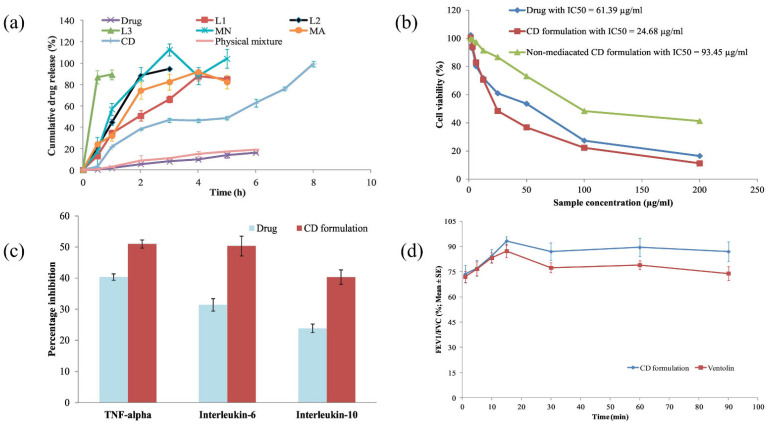
Design and characterization of emulsified spray-dried ALG microparticles as a carrier for the dually acting drug roflumilast (**a**) In an ethanolic phosphate buffer saline solution (30% *v*/*v*; pH 7.4), release patterns of roflumilast and emulsified spray-dried ALG microparticles were studied. (**b**) The effects of roflumilast, a medicated CD formulation, and a non-medicated CD formulation on the proliferation of A-549 tumor cells. (**c**) TNF-alpha, interleukin-6, and interleukin-10 levels were reduced in A-549 tumor cells by roflumilast and CD formulation. (**d**) FEV1/FVC%—time curve in healthy human volunteers upon inhaling the chosen CD formulation vs. Ventolin^®^ HFA. Reproduced with permission from [177], Copyright Elsevier 2018.

Drugs administered to the lungs to treat COPD and cancer suffer from low bioavailability due to distribution to other body tissues, thus requiring multiple dosing and increasing the risk of adverse effects [176]. Mahmoud et al. formulated microparticles based on ALG using the emulsified nano spray-drying process for the pulmonary carrier of roflumilast, a selective inhibitor of the phosphodiesterase-4 enzyme in lung cells. Isopropyl myristate was used as an oil, Tween 80 was used as a surfactant, and calcium beta-glycerophosphate was used as a cross-linking agent. The developed microparticles were assessed based on encapsulation efficiency, particle size, and in vitro release of actives. The aerodynamic data showed that the actives could be deposited deep into the lungs at the bronchi or bronchial tube (as shown in Figure 7a). The formulation with the spherical-shaped microparticles swelled within 3 h at pH 7.4. Furthermore, it showed a remarkable cytotoxic effect on A-549 tumor cell lines (2.5-fold decrease in IC_50_ compared to the pure drug) (as demonstrated in Figure 7b) and reduced pro-inflammatory cytokines (TNF-alpha, IL-6, and IL-10) in contrast to pure actives (as exhibited in Figure 7c). In addition, CD-based microparticles showed higher sustained bronchodilation on human volunteers than marketed Ventolin^®^HFA (as shown in Figure 7d). Thus, CD-based microparticles could be a propitious approach for delivering roflumilast in humans [177].

The pulmonary route has also administered anti-cancer drugs to treat lung cancer due to their faster onset of action and evasion of the first-pass metabolism. One of them, cisplatin, shows toxicity in rat liver, lungs, and kidneys, due to which Alsmadi et al. developed CS-ALG nanoporous aerogel carriers loaded with cisplatin for the treatment of lung cancer using an emulsion–gelation method followed by supercritical fluid extraction. The drug exhibited excellent drug loading (76%) while retaining its crystal structure and gave a sustained release of over 6 h. The cisplatin aerogels, thus prepared, decreased the lung toxicity in rats and prevented weight loss [178].

In another investigation, Iglesias et al. prepared ALG aerogel by thermal inkjet technology followed by supercritical drying to encapsulate salbutamol sulfate, which is used to treat asthma attacks and COPD. The current pulmonary drug delivery system suffers from limitations in particle size uniformity and the presence of surfactants. This can be potentially used for personalized medicine. However, the gel precursor concentrations severely restrict the printable region. The process was highly compatible with the active ingredient (salbutamol sulfate), having a homogenous texture in the nanoporous range. The SS-loaded aerogel was well deposited in the bronchi and bronchioles owing to their nano-particle size. Thus, these aerogels may pave the way for pulmonary drug delivery and personalized medicine, fulfilling the demands of nanostructured, miniaturized, high-resolution product designs [179].

Liposomes, micelles, and polymeric drug particles have been delivered by pulmonary route using biocompatible, biodegradable, and flexible biopolymers such as CS, ALG, gelatin, and other natural polymers. Blends of CS-ALG nanoparticles and microparticles find their applications in controlled drug delivery systems, with calcium chloride often acting as the cross-linking agent. However, researchers have found that using calcium chloride for pulmonary particles induces inflammatory responses. Hence, Alnaief et al. formulated hybrid ALG-CS aerogels via the emulsion-gelation technique without any cross-linking agent. CS solution (2%) was put into the oil phase containing liquid paraffin and 4% surfactant (Span 80 or Span 85), followed by the addition of 1% ALG solution. The resultant hybrid particles were isolated from the oil phase by the process of centrifugation. The pore liquid was replaced with ethanol by a solvent exchange method. The prepared aerogels were later dried with supercritical carbon dioxide. The polymer addition order throughout the gelling procedure and the kind of surfactant employed greatly affected the properties of the hydrogel. Samples prepared using span 85 showed high positive zeta potentials (35.4 ± 5.37 mV), indicating that CS was surrounding the ALG core, whereas those prepared using span 80 showed high negative zeta potentials (−2.15 ± 3.70 to −5.98 ± 5.37 mV), suggesting that ALG was surrounding the CS core. The particle size ranged from 70 nm to 4.17 µm, whereas the aerodynamic diameter ranged from 0.17 to 2.29 µm [180]. Thus, the entire results revealed the developed system is a promising candidate for pulmonary actives delivery.

For the treatment of cystic fibrosis, a potentially life-threatening disease characterized by mutations in the cystic fibrosis transmembrane conductance regulator (CTFR), antibiotics (colistimethane sodium) are generally prescribed. Due to the loss of Cl-channel activity, the lungs become more susceptible to bacterial infections. Of these, Pseudomonas aeroginosa survives the antibiotic treatment and emerges as the predominant infecting organism over time [181,182,183]. Tobramycin is the drug of choice for such infections that are deteriorating with regular colistimethane sodium. One issue associated with tobramycin therapy is the non-compliance of patients due to twice-daily dosing, which leads to the failure of antibiotic treatment. Another concern is the potential for ototoxicity and nephrotoxicity associated with nearly all aminoglycosides, although there is less evidence of ototoxicity and nephrotoxicity in clinical trials with inhaled tobramycin [184,185]. Hence, Hill et al. developed ALG/CS particles using CaCl_2_ as the crosslinker. During the optimization studies, increasing the concentration of cations in the formulation resulted in a more significant aggregation of the particles. The optimal formulation was ALG: CS: tobramycin 9:1:1.5, giving high drug loading and narrow size distribution. Tobramycin release was evaluated, depicting a biphasic release with 18.9% of the entrapped drug released within 24 h. In vitro antibacterial studies showed that the drug-loaded optimal formulation showed a dose-dependent activity against P. aeroginosa with a MIC of 6.25 µg/mL, while the unloaded formulation demonstrated no action. Tobramycin alone had a MIC of 1.5 µg/mL, possibly due to the drug diffusion rate. The Zeta potential was found to be 2.16 ± 0.07% mV and the %EE was 44.5%. SLPI conjugation enhanced the mucoadhesive properties [186]. Hence, the prepared system could help deliver drugs via the pulmonary route.

Moreover, investigators have developed various formulations to intensify pulmonary DDSs. For example, SA/CS/Tween 80/rifampicin [187], ALG/poly(N-isopropyl acryl amide (PNIPAAm)/theophylline [188], ALG/ciprofloxacin [189], ALG/CS/lapazine [190], ALG/CaCO_3_/levofloxacin/DNAse [191], ALG/HA/naproxen [99], ALG/CS/DOX/paclitaxel [192], ALG modified PLGA nanoparticles/amikacin/moxifloxacin [193], ALG particles encapsulating live Bacille Calmette– Guérin (BCG) and *Mycobacterium indicus pranii* (MIP) [194], CS/ALG/BSA gel/DOX [195] are some of the recent formulations showing excellent delivery of drugs via pulmonary delivery.

### 6.4. Vaginal Drug Delivery

Lately, the vagina has been considered a potential administration route replacing the parenteral route for actives delivery, bestowed with systemic effects that cannot be favorably administered per os due to the hepatic or GI degradation or to the commencement of adverse consequences in the GI. The significant constraint of such an administration route is described by the physiological elimination mechanisms, which are active in the vagina’s lumen and are responsible for an unsatisfactory residence time of the conventional formulated system at the targeted site, following an unsteady actives dissemination onto the mucosa [196]. The vagina, a prominent female reproductive organ, allows for local and systemic actives delivery due to its large surface area and high blood supply. However, it is also associated with variations in the size of the endometrium during menstruation, which may alter the drug absorption properties [197,198].

Vaginal dosage forms should ideally be easy to administer, requiring fewer doses, and patient-compliant. However, it is challenging to formulate small water-soluble drugs through the vaginal route. Hence, Meng et al. prepared spray-dried microparticles (MPs) using thiolated-CS coated SA by the layer-by-layer method for the vaginal delivery of HIV microbicides. They were studied for their cytotoxicity and pre-clinical safety on human vaginal (VK2/E6E7) and endocervical (End1/E6E7) epithelial cell lines and in vivo on female mice. The outcomes showed that TCS-coated Ag-based multilayer microparticles had 20–50 fold more adhesion than native AGMPs [199]. Hence, the developed formulation could be useful for vaginal drug delivery.

Urogenital infections affect about a billion women worldwide. It is caused due to *Escherichia coli* and other enterobacteria commonly found in the vaginal tract. The chances of UTIs are increased in post-menopausal women, which account for 25% of all bacterial infections. Cystitis, the most common UTI, usually affects young women, with *E. coli* being the cause of most diseases. The treatment of UTIs is still a challenge because of frequent recurrence, the association of co-morbidities, and high prevalence. Cefixime, a third-generation cephalosporin, is usually given for such infections. However, its poor water solubility, limited oral bioavailability, and incomplete absorption limit its use. Hence, Maestrelli et al. developed a bioadhesive vaginal dosage form of cefixime that can overcome many drawbacks. They prepared CS-coated CA microspheres using an ionotropic gelation method, and their mean weight, diameter, %EE, and loading capacity were evaluated. All MS batches showed prolonged adhesion greater than 2 h on the excised porcine vaginal mucosa; %EE enhanced with increasing drug concentration. In vitro studies revealed the direct relationship between CFX drug release and % inhibition in *E. coli* metabolic activity [200]. Thus, the overall results demonstrated the developed system as an auspicious candidate for vaginal delivery of actives.

Despite having several benefits, for example, enormous surface area, evasion of the first-pass metabolism, and the ability to optimize drug absorption for systemic effects, it also has certain drawbacks, such as the restoration action on vaginal fluids and its acidic environment (pH 4.0–4.5), which hampers the local delivery of drugs due to low residence time and stability. Hence, Ferreira et al. formulated CA hydrogels based on the earlier formation of polyelectrolyte complexes (PECs) for the vaginal delivery of polymyxin B. First, PECs were formed between ALG and PMX, ensued by cross-linking with calcium chloride. The aforementioned system displayed a pore size of between 100–200 μm and adequate syringability; in vitro tests demonstrated mucoadhesiveness. The drug release was found to be pH-dependent with a sustained release of six days. A burst release was noticed at pH 7.4, and the drug was released by anomalous transport. At pH 4.5, actives release followed the Weibull model and actives transport was through Fick’s diffusion [201]. Thus, the formulated system could be suitable for vaginal drug delivery.

Vulvovaginal candidiasis caused by the fungus Candida albicans is generally treated using azole antifungals like fluconazole given orally. However, fluconazole is reported to have severe side effects like nausea, vomiting, diarrhea, and abdominal pain [202,203]. Hence, Darwesh et al. developed vaginal inserts using PEC based on anionic ALG and cationic CS. The mucoadhesion was highest for Na–Alg-based vaginal inserts (pKa 3.21) as compared to carbopol (pKa 5.0) because the mucoadhesion depends on the no. of hydrogen bonds (-OH, -COOH) concerned in the mucoadhesion interaction. The ALG: CS (5:5) PEC revealed controlled release of fluconazole (RE_6h_ ranged from 56.46 ± 3.42 to 79.38 ± 3.42%), good mucoadhesion, and therefore suitable vaginal retention. Moreover, it demonstrated excellent antifungal action against Candida albicans both in vitro (MIC for fluconazole vaginal inserts was 31 ± 0.4 mm and that of fluconazole solution was 22 ± 0.4 mm) and in vivo (complete healing after seven days in rats) with reduced inflammatory cells [204]. Thus, the overall results depicted that the formulated system could be a promising candidate for vaginal drug delivery.

In another investigation, Soliman et al. developed an in situ thermosensitive bioadhesive gel for the vaginal delivery of sildenafil citrate as a prospective therapy for endometrial thinning caused by the administration of clomiphene citrate for ovulation initiation in women with type II gonadotrophic anovulation. They were developed using various grades of Pluronic^®^ (PF-68 and PF-127) grades, into which mucoadhesive polymers such as SA and hydroxyethylcellulose were incorporated in different concentrations. The thermosensitive gels were developed by the cold method. The acceptable range of T_sol-gel_ of 28–37 °C was achieved by decreasing PF-127 concentration and modulating the addition of PF-68. There was increased gel viscosity and mucoadhesive force when the concentration of Pluronic^®^ was raised, but there was a reduction in drug release rate during in vitro evaluation in a standard semi-permeable cellophane membrane at pH 4.5 using citrate buffer to mimic the vaginal fluid. Due to its rapid swelling property, SA aided the formation of adhesive interaction between ALG and mucosa, which led to increased mucosal retention. The in situ gels substantially enhanced endometrial thickness and uterine blood flow, thus potentially improving conception chances in anovulatory women with clomiphene citrate failure [205].

Researchers also synthesized ALG/CS/P4/Pluronic^®^ F-127/progesterone [206], CS/SA/polycarbophil/metronidazole [207], SA/CS/*α*,*β*-glycerophosphate/Bletilla striata/tenofovir [208], SA loaded with anise/fluconazole β-cyclodextrin inclusion complexes [209], CS/ALG/metronidazole [210], HPMC/SA/abacavir [211], dextran/ALG nanofibers/clotrimazole [212], Polaxomer 407/SA/*Lactobacillus crispatus* [213], ALG/CMC/clove essential oil [214], and ALG/CS/metronidazole [215] formulations which enhanced the vaginal drug delivery.

### 6.5. Nasal Drug Delivery

Nasal actives delivery includes the inhalation of drugs into the extremely vascularized mucosal layer of the nasal epithelium, which ultimately reaches the systemic circulation [216]. The nose has an enormous surface area for actives absorption, quicker onset of action, and evasion of first-pass metabolism [217]. The curative action of the majority of the drugs is impacted owing to the BBB’s existence. Therefore, nasal route delivery favors therapeutics straightaway reaching the central nervous system (CNS). Furthermore, nasal delivery of actives shows benefits from brain-targeting, fewer side effects, and easy administration [218].

Rhinosinusitis, inflammation of the nasal cavities characterized by nasal polyp growth, typically extending beyond the nasal valve, is generally treated using nasal corticosteroids since oral ones show systemic side effects. However, its long-term use causes an increase in intraocular pressure and changes in serum cortisol levels. Hence, Dukovski et al. formulated dexamethasone-loaded lipid/ALG nanoparticles dispersed in pectin solution based on an in-situ gelling process that undergoes a sol-gel transformation after contact with the Ca^2+^ ions in the nasal mucosa. The in vitro biocompatibility experiments utilizing colon carcinoma Caco-2 cell lines suggested no effect on cell viability; however, in-vivo studies are required to confirm this observation [219].

**Figure 8:**
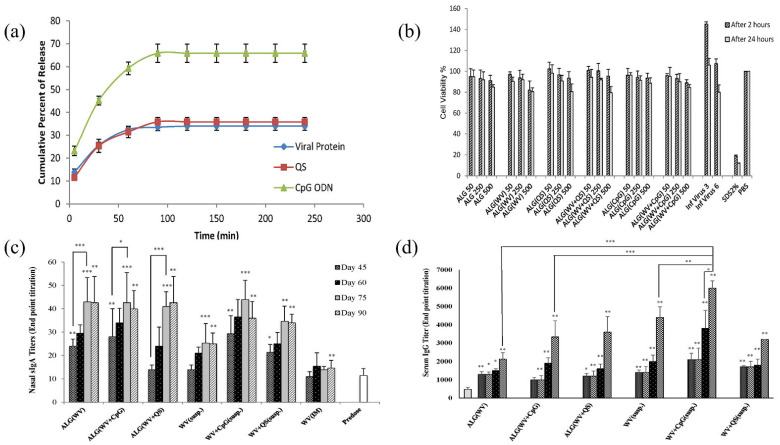
Preparation, characterization, and immunological evaluation of ALG nanoparticles loaded with whole inactivated influenza virus: Dry powder formulation for nasal immunization in rabbits (**a**) % of viral protein, QS, and CpG ODN released in vitro from ALG NPs over four hours. (**b**) After 2- and 24-h exposure with varying concentrations of each formulation, the effect of ALG NPs and influenza virus suspension on cell viability in Calu-6 cell lines (**c**) HAI antibody titers in each vaccination group on day zero (control), day 45 (after prime dose), day 60 (after the second dose), day 75 (after the third dose), and day 90 (after the final booster) (**d**) IgG titers in blood samples taken from each vaccinated cohort on days zero (negative control), 45 (prime dose), 60 (second dose), 75 (third dose), and 90 (after the final booster). * means *p* < 0.05, ** means *p* < 0.01, *** means *p* < 0.001. Reproduced with permission from [220], Copyright Elsevier 2019.

It becomes feasible to administer vaccines in dry powder form during influenza outbreaks due to their ease of transport, patient compliance, and stability. As compared to conventional vaccines, dry powder vaccines provide both humoral and cellular immunity adequately. Thus, Dehghan et al. formulated ALG NPs using the ionotropic gelation method encapsulating whole inactivated influenza viruses and administered them to rabbits, which showed significantly higher IgG titers when short single-stranded cytosine triphosphate and guanine triphosphate-containing synthetic oligodeoxynucleotides (CpG ODN) were used as the adjuvant (as exhibited in Figure 8c,d). ALG NPs released 65.87% of CpG ODN, 35.68% of QS, and 34.00% of influenza antigen within 4 h (as displayed in Figure 8a). In addition, the XTT test demonstrated safety for in-vivo applications (as shown in Figure 8b), making the formulation useful for nasal drug delivery [220].

In a different study, Rao et al. formulated the anti-Parkinson drug Ropinirole as a thermoreversible in situ nasal gel. The rationale for the nasal delivery of Parkinson’s patients is that it becomes difficult for them to swallow oral solid dosage forms due to muscle rigidity. It was prepared by the cold method using PF 127 and HPMC K4M as the thermoreversible polymer. They observed an astounding five-fold enhancement in the bioavailability of ropinirole in contrast to intravenous drug delivery. Furthermore, in vitro evaluation of sheep nasal mucosa demonstrated that the in situ gel had a more protective impact on nasal mucosa than the plain drug, which caused mucosal damage [221].

In another investigation, Youssef et al. developed SA nanoparticles for the anti-migraine drug Almotriptan utilizing the w/o/w double emulsion solvent evaporation procedure. It is a water-soluble drug; hence, SLNs were prepared to help pass the drug through the lipophilic BBB. Poloxamer 407 was used as a stabilizer and evaluated for its physicochemical properties by combining various mucoadhesive polymers, for example, SA, sodium CMC, and carbopol. In addition, the gelling temperature, gelling time, viscosity, gel strength, pH, %EE, and in vitro mucoadhesion were evaluated [222]. The overall results demonstrated the prepared formulation to be suitable for nasal drug delivery.

Vaccines typically include adjuvant substances to enhance the humoral or cellular response to an antigen. The most common antigen, aluminum, instigates a Th2 antibody response; hence, aluminum-based vaccines are unsuitable for intracellular pathogens and chronic diseases. Currently, usable vaccines through the intramuscular (i.m.) or subcutaneous (s.c.) routes fail to elicit a mucosal immune response. Nasal immunization, a relatively simple, non-invasive route, can provide mucosal immunity [223,224,225,226,227]. However, an acceptable mucosal immune response depends on the mucociliary clearance, the tolerogenic character of the mucosal epithelium, and the huge size of the antigen. To circumvent this problem, the antigens can be incorporated into mucoadhesive polymeric NPs, extending the duration of antigen residence and conferring protection against enzymes [228,229]. Hence, Mosafer et al. developed SA-coated CS and trimethyl CS nanoparticles incorporated with the PR8 influenza virus for nasal administration. The zeta potential was −29.6 mV. The vaccine was evaluated in BALB/c mice, wherein the PR8-TMC-ALG formulation manifested a more excellent IgG2a/IgG1 ratio than PR8-TMC, PR8-CHT, and PR8-ALG. Thus, ALG-NPs could be used as immunoadjuvants for nasal immunization. The ALG-coated NPs generated an excellent immune reaction compared to uncoated NPs [230].

The recent nasal formulations include SA/Polaxomer 407/gellan gum/timosaponin BII [231], SA in situ gels based on agomelatine (AGM) [232], ALG based magnetic short nanofibres 3D composite hydrogel encapsulating human olfactory mucosa stem cells [233], OA-dopamine conjugate [234], ALG nanoparticles/venlafaxine (VLF-AG-NPs) [235], *Mycobacterium bovis* Bacille Calmette-Guérin (BCG)-loaded microparticles using ALG/CS [236], ALG/CS/attenuated Androctonus australis hector (Aah) venom [237], CS/ALG nanoparticles/SpBMP-9 (growth factor) [238], ALG/trimethyl CS liposomes/lipopeptide subunit vaccine [239], SA microspheres/*Lactobacillus casei* [240].

### 6.6. Transdermal Drug Delivery

Transdermal drug delivery relates to the delivery of actives via the skin for systemic or local absorption. They are advantageous over the conventional administration routes due to their capability to deliver controlled release of drugs, reducing the first-pass metabolism, less systemic side effects, effective control of drug plasma profile, and patient compliance [241]. However, its applications are limited owing to the extensive skin hindrance, particularly the stratum corneum. Consequently, few actives can penetrate the skin and reach the blood at a therapeutic concentration [242].

Insulin is usually given hypodermically via s.c. injection for the therapy of diabetes mellitus. But it generates biohazardous waste; furthermore, patient compliance is also a challenge, especially in children. Several approaches have come into the picture to resolve the issue, such as inhalational microparticles, oral administration of nanoparticles, and needle-free high-pressure injection systems [243,244,245]. Among these, transdermal patches have attracted much attention owing to minimal pain and tissue injury [246,247]. In this context, Yu et al. fabricated a dissolving polymer microneedle patch comprising 3-aminophenyl boronic acid-modified ALG (ALG-APBA) and HA that can quickly dissolve in the interstitial fluid of the skin. Alginate was chemically modified by adding 3-aminophenyl boronic acid to form ALG-APBA, and HA was crosslinked to form MNs in the presence of Ca^2+^ ions. The MNs thus prepared were evaluated for their mechanical strength, degradation, ex vivo skin insertion, stability of insulin, in vivo transdermal delivery to SD rats, and pharmacokinetic and pharmacodynamic activity in SD rats. In addition, the resistance of MNs to static and dynamic forces was calculated, and it was found that no change in the tips was observed even after the addition of 10–100 g weight on the microneedle patch, with no breaks in the needle after the addition of 500 g, demonstrating its excellent mechanical strength. The encapsulated insulin has comparable pharmacological activity to an s.c. injection with the same insulin dose, with RPA and RBA at 90.5 ± 6.8% and 92.9 ± 7.0%, respectively [248]. Overall results made the aforementioned system advantageous for nasal drug delivery.

In a different investigation, Lefnaoui et al. fabricated transdermal films based on ALG-CS PECs for the antiasthmatic drug ketotifen fumarate (KF). Polyethylene glycol was used as a plasticizer, and Span 20, and Tween 80 were utilized as permeability enhancers. The films were prepared by the film casting method and evaluated for their uniformity in weight, thickness, folding endurance, loss of moisture, and absorption of moisture. Furthermore, actives release and permeation through the rat abdomen mounted on the Franz diffusion cell were characterized. The transdermal films made by a 1:1 ratio of CS and ALG yielded smooth, flexible, strong, bioadhesive, biocompatible films. The drug release research showed that the KF was released in a controlled way over a long period (99.88% release after 24 h) to treat asthma, allergic rhinitis, and conjunctivitis [249]. The results revealed that the prepared formulation is favorable for transdermal drug delivery.

In another study, Abebeet et al. developed a self-adhesive hydrogel for strain-responsive transdermal delivery using gallic acid (GA) modified ALG as the mucoadhesive polymer. The model drug, caffeine, was encapsulated in the hydrogels prepared by the one-pot synthesis method. The diffusion kinetics were controlled by Fickian diffusion. The developed hydrogels had a 25% increase in tensile strength and twice the transdermal release of the drug. A powerful adhesion of 100 kPa was reported on a glass substrate. A close to 800% strain was observed, and it was attributed to the free movement and adhesion of ALG and polyacrylic acid. It could withstand body movement as well as skin stretching when tested on human skin. Thus, GA hydrogel seems to be a promising route for strain-controlled TDD [250].

In another investigation, Abnoos et al. prepared a CS-ALG nanocarrier for the transdermal delivery of pirfenidone (PFD) in idiopathic pulmonary fibrosis, a disease characterized by progressive dyspnea and pulmonary function loss [251,252]. CS-SA nanoparticles were developed by the pre-gelation technique, and the drug Pirfenidone, an anti-inflammatory and antifibrotic agent, was encapsulated with 94% efficiency. The prepared nanoparticles were evaluated using SEM, TEM, and DLS. These studies showed that particle morphology was spherical with an average size of 80 nm. FTIR spectra observed the complex formation between CS and ALG. The drug release studies show that PFD had undergone an initial burst release of 12% after 5 h and was later released in a sustained manner for 24 h. Ex vivo studies revealed that skin permeation of PFD was improved by using CS-SA nanoparticles compared to standard PFD solution. The permeation was also observed through fluorescent microscopic images labeled with FITC. SEM studies reveal that the nanoparticles could remain stable for six months [253]. Thus, the results proved that the prepared formulation is useful for transdermal drug delivery.

In another investigation, Anirudhan et al. formulated bio-polymer matrix films acquired from CMC, SA, and PVA to transdermally deliver diltiazem, a calcium channel blocking drug used for cardiac failure. The oral bioavailability of DTZ is only 30–40%. Hence, alternative routes of drug delivery are being explored. Since diltiazem (DTZ) is hydrophilic in nature, a hydrophilic matrix consisting of polyethylene glycol coated vinyl trimethoxy silane-g-CS (PEG@VTMSg-CS) with matrices like Na-ALG, CMC, and PVA was developed. A dispersion of the matrix was prepared to avoid the undesirable effect of shrinkage of the polymer network when the drug is being eluted out of the matrix. The drug release studies were based on Franz diffusion cells. SA films showed more significant swelling than PVA films. The ALG films displayed a thickness value of 0.052 ± 0.01 mm and a water permeation of 0.07 g cm^2^/24 h. DTZ permeation was less due to less film thickness. The in vitro skin penetration research of DTZ on rat skin revealed the effectiveness of the films with more than 49% viability in HaCaT and PBMC cell lines with no histological alterations on the skin. The present formulation could deliver 70% of the drug in 24 h [254]. Thus, the overall results demonstrated that the system is promising for transdermal drug delivery.

The currently investigated formulations include ALG/PVA/ciprofloxacin electrospun composite nanofibers [255], SA/propylene glycol/metoclopramide films [256], SA/glucosamine sulphate [257], ALG/maltose composite microneedles/insulin [258], ALG/CS/rabeprazole [259], oleic acid/SA/Na CMC/2,3,5,40-tetrahydroxystilbene 2-O-β-D-glucoside (THSG) [260], SA/CS/piplartine [261], SA/polyvinyl alcohol/quercetin [262], ALG/Hidrox-6/Hydroxytyrosol [263], sodium L-cysteine ALG/isopropyl myristate/ropinirole hydrochloride [264], ALG coated aminated nanodextrin/CS coated folate decorated aminated β-CD nanoparticles/curcumin/5-flurouracil [265] which intensified transdermal drug delivery.

### 6.7. Mucosal Drug Delivery

In order to evade parenteral routes and beat biological impediments, one more DDS, specifically the mucosal delivery system, appeared in the limelight. Mucus gel layers line the vagina, lungs, and gastrointestinal tract (GIT). GIT represents a complicated condition, including several digestive enzymes and a changeable pH (acidic pH-stomach and basic pH-intestine). Different drugs necessitate being guarded against the circumstances referred to above to evade degradation by proteolytic enzymes. These pharmaceuticals exhibit inadequate permeability in gastric mucosa and also bear the first-pass effect. To preserve the actives of interest facing such conditions, ALG serves as one of the most satisfactory polymers with mucoadhesive characteristics and improved permeation results, and acts as a defensive envelope for such actives through mucosal delivery [266]. Mucoadhesion refers to the capability of materials to bind to the mucosal membrane and offer some retention. Mucoadhesive drug delivery provides various benefits over other routes of administration, for example, faster distribution to the local blood vessels, avoidance of first-pass metabolism, reduced dose frequency, and rapid relief [267]. Thus, the formulated system might be helpful for mucoidal drug delivery.

Oral cancer, one of the most common cancers, is usually treated with surgery and chemotherapy [268], but they have the drawback of alteration of facial morphology followed by surgical procedures. Hence, to provide an alternative approach, Shtenberg et al. formulated a mucoadhesive ALG/liposome paste. DOX-loaded liposomes were prepared, and ALG-fluorescein was synthesized using FITC and ethylenediamine; on homogenization, a cross-linked hybrid paste was formed. It showed the liposome’s sustained release from ALG cross-linked paste (release index of 8). Furthermore, in vitro toxicity studies on human cell lines acquired from tongue SCC (CAL 27) showed viability of 15% after 48 h. Thus, polymers released from the liposomes were active and effective. Therefore, this approach could be used for potential oral cancer therapy [269].

**Figure 9:**
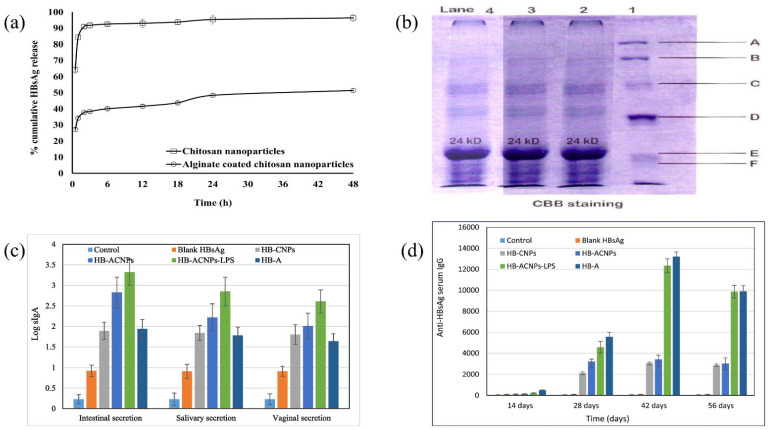
Lipopolysaccharide-derived ALG coated Hepatitis B antigen-loaded CS nanoparticles for oral mucosal immunization (**a**) Release profile of produced nanoparticles in vitro. (**b**) HBsAg release as determined by SDS-PAGE: Lane 1: Molecular weight markers; Lane 2: HBsAg solution; Lane 3: HBsAg loaded CS nanoparticles, Lane 4: HBsAg-loaded ALG coated CS nanoparticles produced from LPS. (**c**) The levels of sIgA in the fluid secretions of mice immunized with different formulations. (**d**) Anti-HBsAg IgG levels in mice inoculated orally with various formulations. Reproduced with permission from [270], Copyright Elsevier, 2020.

Oral mucosal vaccine delivery has the disadvantage of significant enzymatic degradation of the antigen in the GI tract, and these have difficulty in uptaking from the intestine. To overcome this issue, HbsAg-loaded CS nanoparticles with an ALG coating were prepared by Saraf et al., in which lipopolysaccharides acted as an adjuvant. It showed elevated levels of sIgA in intestinal (3.32 mIU/mL) secretions compared to non-ALG coated HB-CNPs (as displayed in Figure 9c). Thus, ALG-coated CS NPs (CNPs) could entrap HbsAg effectively (as shown in Figure 9b). The release profile of protein showed that 84% of the drug was released at 0.5 h. owing to CNPs’ loose binding that led to increased desorption at the acidic pH, but 95.5% of the drug was released at 48 h (as displayed in Figure 9a). Coating with ALG stabilized increased the stability of CNPs and provided a sustained release of 51.42% after 48 h. In addition, the ALG-coated NPs adhered better than uncoated NPs. The cell viability of RAW 264.7 cell lines was 70.45%; nevertheless, the cell viability decreased to 66.45% after 48 h. This time-dependent decrease in cytotoxicity could be due to contact between positively charged nanoparticles and negatively charged membranes. Mucosal M-cell-specific ALG-coated CS nanoparticles elicited a significant immunological response in mice (as demonstrated in Figure 9d). Hence, overall results showed the aforementioned system is favorable for mucosal drug delivery [270].

In another investigation, Ghumman et al. utilized linseed mucilage-ALG mucoadhesive microspheres loaded with Metformin HCl developed by an ionotropic gelation procedure for mucosal actives delivery. Drug encapsulation efficiency was up to 92% with sustained release for 12 h. The results revealed that an optimized formulation (FM-4) could furnish sustained release for up to 12 h and hold the level of blood glucose by regulating and enhancing the absorption of metformin systemically. Therefore, LSM, a natural emerging mucoadhesive agent, proved to be preferable for controlled release mucoadhesive microspheres designed for oral utilization [271].

In periodontitis, there is inflammation and destruction of tooth tissues, and the pathogen forms a biofilm around the inflamed tissues. Currently, treatment approaches include mechanical removal of the biofilm aided by antibiotics whose systemic side effects limit the effectiveness of treatment. Hence, Kilicarslanet al. used clindamycin phosphate-loaded ALG/CS PEC film to overcome this issue. CS and ALG, being oppositely charged biopolymers, form a PEC by cross-linking with each other. It was observed that increased ALG concentrations in the polymer mixture increased adhesiveness, making it valuable for mucosal drug delivery [272].

In another investigation, Gonçalves et al. developed highly porous ALG/carrageenan aerogel nanoparticles for drug delivery of powdered model drugs quercetin and ketoprofen. Biopolymer aerogels have low toxicity and are biocompatible, with large surface areas with accessible pores or drug loading. The emulsion gelation method was used to produce the hybrid biopolymer nanoparticles, followed by drying using supercritical CO_2_. Pectin and ALG had better interaction, with an increased degree of cross-linking between the two polysaccharides giving NPs of higher specific surface area (>300 m^2^ g^–1^) and lower shrinkage. The drug-loading capacity was 17 ± 2% for ketotifen and 3.8% for quercetin. The cytotoxicity test using the MTS assay on human carcinoma Caco-2 cell line showed almost 100% cell viability, thus demonstrating the prepared nanoparticles to be highly non-toxic. The drug release from ALG/κ-carrageenan aerogel was quicker than from ALG/pectin [273]. Thus, the developed system was found to be suitable for drug delivery.

In another research, Martín et al. developed effective antifungal mucoadhesive drug delivery systems to treat oral candidiasis using ALG microspheres containing the drug nystatin. Oral candidiasis is caused by the opportunistic pathogen Candida albicans, especially in immunocompromised, diabetic patients undergoing chemotherapy. Nystatin, a polyene antifungal antibiotic, is indicated to treat mucocutaneous fungal infections caused by C. albicans. However, nystatin possesses a huge lactone ring and numerous double bonds, which render it amphiphilic and amphoteric and thus render its formulation difficult. Consequently, it has been formulated as micellar gels, liposomes, intralipids, niosomes, and various other dosage forms [274,275,276]. Thus, Martin et al. developed nystatin microspheres based on the emulsification/internal gelation procedure with some modifications [277]. Evaluation of particle size, zeta potential, swelling behavior, loading content, encapsulation efficiency, rheology, mucoadhesive force, drug release studies, antimicrobial activity, and in vivo studies were carried out. It was found that the mean size of the microspheres was 85–135 um, and the viscosity was that of a Newtonian fluid. The drug release was in two steps; first, an initial burst release was followed by a more sustained drug release, with about 60% of the drug released in the first 2 h. The prepared microspheres showed marked antifungal activity with a drastic reduction in C. albicans colonies. In vivo research on the buccal mucosa of experimental animals demonstrated that the retained amount, which is 4 to 6 times higher than the MIC, was enough to elicit the therapeutic response [278].

Researchers have also formulated lysozyme mucoadhesive tablets/Ca^2+^ cross-linked ALG with HPMC [279], silybin/nanocrystals-in-microspheres PEC/ALG/CS [280], co-delivery of ketorolac and lidocaine/polymeric wafers/2:1 SA:PVP −25 and 10% glycerol [281], HPMC/SA/nicotine [282], ALG modified with maleimide-PEG/ibuprofen sodium [283], silicone sheet/dexamethasone/ALG [284], ALG/sterculia gum/citicoline [285], ALG/ghatti gum/montmorillonite/flubiprofen [286], pectin/ALG/repaglinide [66], polyacrylamide-g/locust bean gum/ALG/ketoprofen [287], CS/CA/*Lactobacillus casei* [288] for improved mucosal actives delivery.

### 6.8. Intravenous Drug Delivery

ALG, owing to its biocompatibility and safety in vivo, has been explored for intravenous drug delivery in order to increase the bioavailability of drugs that the conventional oral route cannot deliver.

Hydrophobic medicines can be entrapped in hydrophilic nanoparticles like CA and delivered to their site of action. Curcumin and resveratrol are both polyphenolic molecules found in nature that have anti-cancer properties. Their low water solubility and bioavailability unfortunately limit their therapeutic utility. In a study by Saralkar et al., the emulsification and cross-linking procedures were used to make curcumin and resveratrol-based CA nanoparticles. Particle size, zeta potential, moisture content, physicochemical stability, and %EE were all measured in the nanoparticles. For the combined estimation of curcumin and resveratrol, the UPLC methodology was designed and validated. The in vitro efficiency of ALG nanoformulation on DU145 prostate cancer cells was investigated. Curcumin and resveratrol had entrapment efficiencies of 49.3 ± 4.3% and 70.99 ± 6.1%, respectively. In 24 h, resveratrol had a greater release than curcumin (87.6 ± 7.9% against 16.3 ± 3.1%). Curcumin, both in solution and as nanoparticles, was found to be taken up by cells. Resveratrol was poorly absorbed by cells. On DU145 cells, the drug-loaded nanoparticles cause cytotoxicity. The drug solution was more hazardous than nanoparticles at high concentrations. The intravenous administration of the ALG nanoformulation was proven to be safe [289].

Over the last 10 years, multifunctional theranostics have created some intriguing novel possibilities for chemotherapy and tumor detection. In a study by Yang et al., the photosensitizer chlorin e6 (Ce6) and the anticancer medication DOX were subsequently adsorbed onto the magnetic mesoporous silica nanoparticles (M-MSNs) to produce a pH-sensitive drug release and for adsorbing P-glycoprotein short hairpin RNA (P-gp shRNA) for preventing multidrug resistance, ALG/CS polyelectrolyte multilayers (PEM) were constructed on the M-MSNs (M-MSN(DOX/Ce6)/PEM/P-gp shRNA). Upon laser illumination, the nanoparticles with a mean diameter of 280 nm showed a pH-responsive drug release profile and increased singlet oxygen formation in tumor cell lines. The delivery mechanism only released 12 percent Ce6 after 30 h at pH 7.4 but >95 percent after 36 h at pH 4.0. In terms of DOX release, roughly 30% of it was released from M-MSN(DOX/Ce6)/PEM nanocomposites in 32 h at physiological pH (7.4), compared to a 46% release of DOX at pH 4.0. The multifunctional nanocomplexes greatly boosted apoptosis in vitro, as demonstrated by the CCK-8 assay and calcein-AM/PI co-staining. Using cancerous Balb/c mice for the animal studies, researchers used a combination of photodynamic treatment and chemotherapy to achieve a synergistic anti-cancer activity in vivo. Additionally, the cores of the bifunctional Fe_3_O_4_-Au nanoparticles in the multifunctional nanocomplexes allowed dual-modal MR and CT imaging, revealing high uptake into tumor-bearing animals via intravenous injection. This study demonstrates the excellent performance of magnetic mesoporous silica nanocomposites as a multipurpose delivery system for imaging-guided cancer synergistic therapy [290].

For cancer treatment, nanocarrier drug delivery systems (NDDSs) have received more attention than traditional drug delivery methods. The rapid evacuation of activated macrophages from the bloodstream, however, hinders efficacy. In a study by Wang et al., glycyrrhizin (GL) was loaded into ALG nanogel particles (NGPs) to create a versatile delivery mechanism to reduce activated macrophage clearance and improve anticancer activity with GL and DOX combined therapy. GL-ALG NGPs could not only prevent eliciting macrophage immuno-inflammatory responses, but they could also reduce macrophage phagocytosis. DOX/GL-ALG NGPs increased DOX bioavailability by 13.2 times compared to free DOX in the blood. The use of mice with normal immune systems instead of nude mice in the construction of tumor-bearing mice further revealed that NGPs are biocompatible. In vitro and in vivo, GL-mediated ALG NGPs had an exceptional hepatocellular carcinoma targeting effect [291].

Arsenic trioxide (ATO) is efficacious in managing acute promyelocytic leukemia (APL) and late-stage primary hepatic cancer, although it has serious adverse effects. Hence, Lian et al., in order to address these issues, synthesized red blood cell membrane-camouflaged ATO-loaded SA nanoparticles (RBCM-SA-ATO-NPs, RSANs). Ion crosslinking was used to make ATO-loaded SA nanoparticles (SA-ATO-NPs, SANs), wherein RBCM was deposited over the surface to make RSANs. RSANs had a mean particle size of 163.2 nm with an entire shell-core bilayer arrangement and a 14.31 percent encapsulation efficiency. When relative to SANs, a decreased phagocytosis in RAW 264.7 macrophages was observed RSANs by 51%, and the in vitro cumulative release rate was 95% at 84 h, indicating a notable sustained release. Moreover, RSANs were found to have reduced cytotoxicity than natural 293 cells and to have anti-cancer activity on both NB4 and 7721 cells. In vivo investigations also revealed that ATO can induce minor organ damage, whereas RSANs can minimize toxicity and boost anti-tumor efficacy [292].

Curcumin administration by nanocarriers is an appealing strategy for overcoming its limited bioavailability and rapid metabolism in the liver. Karabasz et al. developed AA-Cur, a blood-compatible ALG-curcumin combination that produced colloidally stable micelles of around 200 nm and displayed high cytotoxic effects against mouse cancer cell lines, as previously demonstrated. In their study, they investigated AA-toxicity and anticancer efficacy in two different animal tumor models. In the first study, C57BL/6 mice were administered with colon cancer MC38-CEA cells subcutaneously. Breast tumor 4T1 cells were administered orthotopically, that is, into the mammary adipose tissue of BALB/c mice in the second study. Investigations of blood biochemistry, histology, morphology, DNA integrity (comet assay), and cytokine screening were used to assess the toxicity of intravenously injected AA-Cur (flow cytometry). The anticancer effects of AA-Cur were determined by comparing the development of colon MC38-CEA- or orthotopically injected breast 4T1 tumor cells in untreated and AA-Cur-treated mice. Four injection dosages of AA-Cur revealed no toxicity, showing that the conjugate is safe to use. The anti-cancer efficacy of AA-Cur was moderate in colon MC38-CEA and breast 4T1 carcinomas [293].

Other applications of ALG in intravenous drug delivery include ALG/deferoxamine conjugates [294], ALG Microparticles/Amphotericin B [295], ALG/poly(amidoamine)/MC3T3-E1 pre-osteoblasts hybrid hydrogel [296], octanol grafted ALG nanoparticles/propofol [297], pH-Responsive ALG/CS multilayer coating on Mesoporous Silica Nanoparticles encapsulating DOX [298], ALG-glycyl-prednisolone conjugate nanogel [299].

### 6.9. Others

The buccal mucosa is an extremely appealing route of actives delivery for actives with low bioavailability, poor gastric stability, and vulnerability to first-pass metabolisms such as proteins and peptides, by carrying the actives straightaway into the bloodstream. The oral mucosa also lacks Langerhan cells, making the oral mucosa tolerant to various allergens [300]. Aphthous stomatitis, a type of inflammation in the intraoral cavity whose causative agent is still unknown, is currently treated symptomatically using corticosteroids. Ambroxol is gaining popularity as an upcoming agent to treat chronic inflammation. Laffleur and Küppers developed a buccal dosage form by anchoring sulfhydryl groups of the amino acid cysteine onto the ALG adhesive backbone, incorporating ambroxol as the antisecretory drug. Mucoadhesive studies show that ALG-SH had an 11.56-fold increase in adhesion time due to the binding of the sulfhydryl group to the cysteine-rich mucus glycoprotein, leading to prolonged residence time in comparison to the weak van der Waals and hydrogen bonding in native polymers. Permeation studies on freshly excised buccal mucosa showed a 1.89-fold increase in permeation of ALG-SH as compared to native ALG. The mechanism of permeation enhancement was by tyrosine kinase inhibition by disulfide bond formation between the sulfhydryl group of the polymer and the cysteine group of the protein. Ambroxol release from ALG-SH showed a 1.4-fold-controlled release compared to native ALG, possibly due to inter and intra-crosslinking formation, thus providing stability. Therefore, sulfhydryl-anchored ALG could be used for the effective therapy of aphthae [301].

Periodontal diseases affect the gums and bones of the teeth due to bacterial infections. The desirable properties of drug delivery systems for periodontal illnesses include low toxicity, biodegradability, and the ability to treat bacterial infections. Hence, Prakash et al. utilized a controlled-release formulation of amoxicillin using PVA/ALG/hydroxyapatite (HAp) films by wet precipitation to treat periodontal infections. SEM studies show HAp NPs were effectively blended and embedded with amoxicillin irrespective of the annealing temperature. The in-vitro analysis, cell viability assay (70%), fluorescent staining, and hemolysis assay provided conclusive evidence for the suitability of this composite film in treatment. It gave a sustained release, with 87% of the actives released by day 10. The swelling ratio was almost 80% for all films annealed at different temperatures. The tensile strength was more significant than the standard PVA/SA patch, and it increased with increasing annealing temperature. The fabricated films exhibit high anti-bacterial action against *Escherichia coli*, *Staphylococcus aureus*, *Enterococcus faecalis*, and *Pseudomonas aeruginosa*. The fabricated films are also highly biocompatible and hemocompatible, as evidenced by in vitro analysis, cell viability studies, fluorescent staining, and hemolysis assay. Annealing at different temperatures ranging from 300, 500, and 700 °C, respectively, gave films that can encapsulate and release the drug at different extents. HAp also helps in the regeneration of damaged bone segments, in addition to its drug matrix properties. Thus, PVA/SA/HAp/amoxicillin films are good candidates for treating periodontal defects, orthopedic implants, and bone grafting [302].

The sublingual route of vaccine delivery is attracting a lot of attention because of the ease of self-administration of vaccines as well as an abundance of antigen-presenting cells in the sublingual mucosa. In contrast to intravenous and subcutaneous vaccines, sublingual vaccines confer mucosal immunity as well against pathogens like SARS, HIV, and HPV [303]. However, they suffer from a lack of adhesion and absorption through the sublingual epithelium. Hanson et al. prepared a biopolymer platform based on mucoadhesive ALG and CMC polymer wafers loaded with HIV gp14 protein for the delivery via sublingual route and protection of protein vaccines. The wafers were prepared by dissolving ALG:CMC polymers along with NaCl in deionized water. Microstructural analysis of the wafers revealed that CMC wafers had huge pores with thick strands, while the pores of ALG were smaller and smoother. This may be due to CMC being phased out of water during freezing while amorphous ALG chains were more flexible, hence more extensive entanglement. In addition, it was found that wafers with high ALG content showed high mechanical stability as well as protection from impairment owing to lyophilization and exorbitant heat, partly due to their network-forming capability and poor crystallinity. In contrast, a large number of CMC incorporated wafers were extremely mucoadhesive to sublingual mucosa tissue and could endure washing, leading to enhanced protein permeation into the tissue. Compared to liquid gp140 solution, the vaccines produced comparable T-cell and B-cell mediated immune reactions in mice and comparable IgA and IgG levels in blood, vagina, and saliva. The optimum formulation (CMC:ALG ratio of 1:1) could be safely stored and carried without a cold chain while also preserving its immunogenicity following vaccination in mice via a sublingual route. Thus, this novel platform could be used for the potential delivery of sublingual vaccines [304].

ALG-based formulations are presently in the limelight owing to their exceptional characteristics. They are researched meticulously to obtain the utmost benefits of drug delivery (shown in Table 2 and Table 3).

## 7. Recent Advances in ALG Formulations in Wound Healing

Skin represents the largest human organ that functions as a protective barrier against dehydration, pathogens, environmental stresses, etc. [335]. Skin injuries can be acute or chronic and can occur in arterial insufficiency, diabetes mellitus, immunological disorders, and other infections. Rarely does complete re-epithelialization happen in repairing skin defects; hence, increased attention is diverted to preparing wound dressings to promote wound healing and reduce scars while also protecting from microbial infections and dehydration at the wound site [336,337].

Wound dressings play a vital part in wound management as they are applied to a variety of burns and wounds to aid in the repair and renewal of injured tissues, as well as to encourage healing and minimize the infection risk. The optimum wound dressing should decrease the recovery period and discomfort, increase tissue regeneration and recovery, absorb excess exudate from the wounds, encourage healing, and prevent infectious complications. For several decades, scientists have aspired to develop a specialized dressing to treat injuries. Traditionally, dressings are made from plant-based fibers, honey, animal fats, etc. Currently, biopolymeric materials are used as wound dressings as they offer unique properties, such as antibacterial, re-epithelializing, antioxidizing, and anti-inflammatory properties, that significantly promote wound healing [338,339].

Recent developments in wound dressings also enable the release of therapeutic agents to restore skin homeostasis and integrity [336]. Biopolymers are attractive wound healing materials as they can maintain a moist wound bed due to water sorption; they can also absorb any tissue exudates and allow oxygen permeation across the wound [340]. Furthermore, because of the hydrophilic nature and structural features of these wound dressings, a sustained release of the encased bioactive substances could be achieved [341]. Both synthetic and natural polymers are useful for wound dressing, including heparin, CS, HA, dextrans, ALGs, and β-glucans, because of their desirable physical and biochemical properties [338,342].

ALG is a biopolymer widely used for tissue engineering applications, particularly in wound healing [343]. It has been used as a food additive for ages; hence, it is considered a biocompatible polymer. It has found applications in tissue regeneration and bioactive delivery because of its biodegradability and slow dissolution in the biological fluids when cross-linked with exchangeable cations. This rate could be adjusted by controlling the oxidation [344] and reducing ALG’s molecular weight [345]. ALG composites are used for wound healing and soft tissue regeneration to strengthen the capacity and material characteristics of native ALG to adapt to different biomedical applications [346,347,348]. Calcium ions are released when water-insoluble CA encounters wound exudates as a function of calcium ions being replaced by sodium ions in bodily fluids, which can operate to achieve hemostasis. The SA-based fibers absorb a massive amount of exudates and transform into a gel-like substance, which maintains the moist barrier on the wound surface [349,350]. Some recent investigations of ALG composites in wound healing applications have been discussed in Table 4.

Diabetes-induced wounds currently have no effective treatment and thus represent a challenge in wound healing. Reports suggest that bacteria-caused inflammations due to the alkaline pH of ulcer wounds and incomplete blood flow to the wounds due to slow angiogenesis may be responsible for delayed diabetic wound healing. Hence, Wang et al. developed a novel, pH-sensitive CA-based hydrogel loaded with protamine nanoparticles and hyaluronan oligosaccharides (protamine NP/HAO CA hydrogel) by the ionotropic gelation method. The loading efficiency of HAO was 85.4 ± 6.25%, and 44.5% of the drug was released in 8 h at pH 3.0. At pH 8.0, which mimics the diabetic wound state, CA hydrogel swelled by the absorption of water, and a faster drug release of NPs and HAO took place. Compared to plain CA hydrogels, the protamine-loaded ones showed improved antibacterial activity against *E. coli* and *S. aureus*, enhanced adherence to wound tissue sites, and absorption of wound fluid into the hydrogels. Wounds closed with rates of up to 96.8% after 14 days without ulceration and festering at the wound site. Re-epithelialization was observed, indicated by the formation of fibroblasts and collagen. Moreover, the composite hydrogels reduced inflammation and encouraged angiogenesis [351].

PECs produced from the polysaccharides ALG and CS at defined flow rates, pH, and agitation speeds give rise to slender, stable, and transparent sponges or films possessing excellent swelling ability in physiological medium. Unfortunately, these PECs have inadequate mechanical properties, which may negatively affect their performance in articulated regions such as knees, shoulders, and elbows. To overcome this, Pires et al. fabricated CS-ALG-based wound dressings with the addition of poly (dimethylsiloxane) to improve the flexibility and sorption capacity in contact with biological fluids. The optimum amount of poly(dimethylsiloxane) per gram of PEC was found to be 0.1 g, wherein the formulation exhibited high stability, a tensile strength of 12 MPa, non-hemolysis, and induction of thrombus formation. Moreover, when beta-carotene and thymol were loaded onto the formulations via the supercritical carbon dioxide impregnation/deposition (SSI/D) technique at 45 °C and 250 bar for 14 h, higher bioactive loading capacities were observed using SSI/D and a high depressurization rate (10 bar/min). A substantial amount of Thy and Bc were preserved in the matrix structure with the SSI/SD method, which functioned as a reservoir system. Thus, the combination of ALG, CS, and silicone gel could be a potentially successful wound dressing in the case of less articulated body sites and low extruding wounds [352].

**Figure 10:**
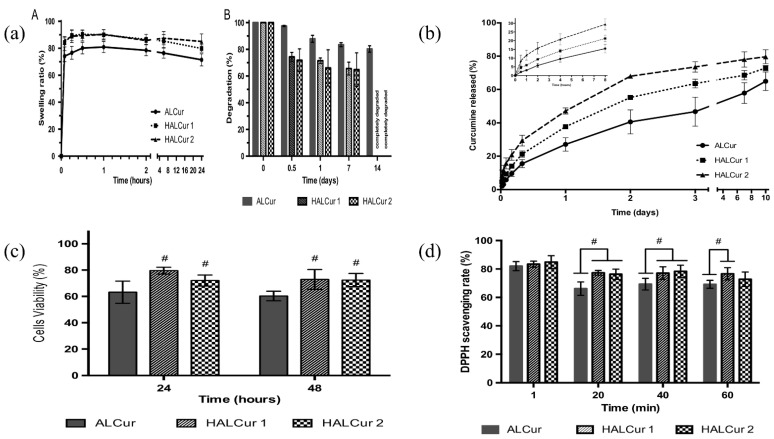
ALG/human elastin-like polypeptide composite films with antioxidant properties for potential wound healing applications. (**a**) swelling (A) and stability (B) graphs of ALCur, HALCur films encapsulating 0.1% curcumin. (**b**) % curcumin release characteristics of ALCur, HALCur films encapsulating 0.1% curcumin. (**c**) Cell viability assay of ALG/HELP composites on human fibroblast cell lines. (**d**) DPPH assay of ALCur and HALCur films indicating antioxidant activities. # *p* <0.05. Reproduced with permission from [353], copyright Elsevier 2020.

The combination of ALG with proteins holds a lot of promise in enhancing the cellular interactions of ALG and for tailoring the biodegradation of the composite materials in tissue regenerating applications. Elastin, a highly flexible protein abundant in the extracellular matrix, is a good candidate for composites because it renders the tissues elastic enough to withstand continuous cycles of deformation/recovery without rupturing. Hence, Bergonzi et al. fabricated ALG/human elastin-like polypeptide (HELP) hybrid films by the solvent casting method with loaded curcumin to provide antioxidant activity. Strong intermolecular hydrogen bonding between the N-H amide bonds of HELP and the carboxylate group of ALG leads to close network formation due to molecular associations, thus giving higher tensile strength and Young’s modulus, which is representative of its higher elasticity. Swelling capacity was higher in HALCur than in ALCur (as shown in Figure 10a). The incorporation of HELP was not only instrumental for obtaining a controlled release of curcumin (as depicted in Figure 10b), which helped in a higher antioxidant effect (as illustrated in Figure 10d), but also in enhancing the cytocompatibility of the final biomaterial, as shown in Figure 10c). With more in vivo studies, it may be possible to design customized bioplatforms for biomedical applications [353].

Full-thickness healing requires extensive healing time, which increases the risks of infections, wound ulcers, necrosis, and even fatal complications. To tackle this, a hybrid hydrogel composed of amine-functionalized fish collagen and OA was prepared by Feng et al. by a simple Schiff base reaction, and antimicrobial peptides bacitracin and Polymyxin B (AC/OSA-PB) were loaded into the hydrogels without the need for catalysts. These hydrogels illustrated modifiable gelation time, stable rheology, and a strain resembling that of human skin. Moreover, they could effectively cause inhibition of *S. aureus* and *E.coli* growth, promoting angiogenesis and cell proliferation in vitro. Similar results were observed in vivo too, with enhanced full-thickness wound healing ability by promoting granular tissue formation and deposition of collagen and accelerating neovascularization and re-epithelialization [354].

In a similar study related to full-thickness wound healing by Chaudhary et al., CA nanoparticles (CA-NPs) were used as hemostatic agents along with antimicrobial AgNPs in a CS-based hydrogel network. Herein, the fresh blood of the subjects was used in the hydrogels to substitute the growth factors required for wound healing. The CA-NPs had mean hydrodynamic sizes of 1037 nm and 120.56 nm, respectively, and a zeta potential of less than −30 mV indicated their negligible electrostatic repulsion tendency. The prepared hydrogels exhibited good spreadability and viscoelasticity. Moreover, they showed bacterial inhibition against both gram-positive and gram-negative strains and also illustrated remarkable scar-free healing in vivo for up to 15 days by aiding in collagen deposition and acting as a protective sheath against microbial contamination for diabetes-induced wounds. Thus, the proposed composite films could be an exciting wound dressing for patients who have chronic diabetes [355].

In another study, Sharma et al. loaded rifampicin into ALG-gelatin fibers through a physical cross-linking reaction by the extrusion-gelation method and then embedded it into transdermal films for wound healing applications. The tensile strength of the fibers was between 2.32 ± 0.45 to 14.32 ± 0.98 N/mm^2^, and the extensibility was between 15.2 ± 0.98% to 30.54 ± 1.08%. The range of moisture absorption was low (up to 14.68%), which is essential for transdermal films. Other parameters such as the swelling ratio and water vapor transmission rate demonstrated the extensive gelation properties of the polymer and the reduced channel pore size. Antibacterial activities against *E. coli* and *S. aureus* were observed for the transdermal films. In vivo animal studies exhibited close to 83 degrees of contraction of the wound, marginally less than the commercial formulation (91.87 ± 3.72%). The drug release from the films followed sustained release; this could be due to sufficient contact of the wound dressing with the wound layer with hair growth signs evident from the 10th day onwards. Thus, the proposed fiber-in-films could be excellent carriers for drug delivery and wound healing purposes [356].

Other studies where ALG composites showed promising results in wound healing include SA/HA films/sulfadiazine/silver nanoparticles [357], ALG/gelatin fibers/curcumin [358], SA/xanthan gum film/pycnogenol [359], ALG/CS/maltodextrin/pluronic F127/pluronic P123/tween 80/curcumin polymeric micelles [360], SA/pectin/cefazolin nanoparticles [361], CA/ibuprofen hydrogels [362], ALG/cannabidiol [363], SA/polyvinyl alcohol/curcumin/graphene [364], ALG/polyhexanide/AgNPs [365], ALG/carboxymethyl CS/Kangfuxin sponges [366].

**Table 4:** Recent investigations of ALG-based composites for wound healing applications.

Type of Wound Dressing Materials	ALG-Based Composite Materials	Applications	References
Nanocomposite hydrogel	ALG/Eudragit	Chronic cutaneous wound healing in diabetic mice	[367]
Hydrogel	ALG/collagen	Drug delivery for skin wounds in trauma patients	[368]
Hydrogel	ALG/Pluronic F127	Drug release-based bleeding wound healing	[369]
Films	ALG	Chronic wound healing	[370]
Hydrogel	ALG/gelatin methacrylate	Wound healing	[371]
Hydrogel	Oxidized ALG	Chronic wound healing in diabetic mice	[372]
Thermoreversible hydrogel	SA/chondroitin sulfate	Drug delivery and diabetic wound healing	[373]
Ion exchange responsive film	ALG/hyaluronate	Drug delivery and skin wound healing	[374]
Films	ALG/pectin	Wound healing for moderate exudates	[375]
Hydrogel	SA/poly(N-isopropyl acrylamide)	Drug delivery and wound healing	[376]
Xerogel	ALG/g-polyethylene glycol methacrylate	Wound healing	[377]
Film	SA/K-carrageenan	Sustained drug release-based topical wound dressing	[378]
Electrospun mat	ALG/polyvinyl alcohol	Wound dressing and real-time evaluation of healing	[379]
Sponges	ALG/CS/HA	Wound healing in full-thickness wounds in rats	[380]

## 8. Conclusions

ALG emerges as a prospective naturally derived biomaterial in NDDSs due to the biocompatible, degradable characteristics and gel-forming capability of ALG. Controlled and targeted actives delivery via ALG carrier can be accomplished via well-created formulation as well as accurate parameters of synthesis concerned with the process of fabrication. Hence, this article provides an extensive review of the recent advances of ALG and its advancement in actives delivery. The most significant characteristics of ALG encompass safety, biocompatibility, and ease of preparation. Due to its biocompatible, biodegradable, and non-toxic characteristics, it is employed in diverse drug-delivery technologies. A significant challenge that persists is the preparation of environmentally friendly procedures for the NPs formation having a narrow size distribution, high mechanical and chemical stability, and practicality to scale up to the volumes of industrial-scale production. Moreover, it is critical to assess the toxic effects on cells, immune response, and biodegradability of such formulations in drug delivery.

As we look towards the future, the ALG-based composites utilized in pharmaceutical applications are possibly going to develop significantly. Although ALG composites are already used clinically for wound healing, they perform quite a passive function. Forthcoming dressings will probably perform a considerably more active role. One or more bioactives that assist wound healing can be loaded into ALG-based dressings, as such gels have displayed usefulness in preserving local conc. of biological factors (for example, proteins) for a prolonged period. In wound healing, and more usually, actives delivery, accurate control over the single vs. multiple drugs delivery or drug release in sustained vs. sequential manner in response to exterior environmental modifications is immensely advisable.

Therefore, investigators are required to modernize the ALG-associated composite’s advancement, and this review is the origin of advice for forthcoming investigation.

## Figures and Tables

**Table 1:** Various methods for the extraction of ALG.

Seaweed Species	Components Used for Extraction	Extraction Yield (% Dry Weight—d.w.)	Characteristics	References
*Sargassum mangarevense* and *Turbinaria ornata*	Formaldehyde-acidification-Na_2_CO_3_-ethanol	*Sargassum mangarevense* (6.0–12.4% d.w.); *T. ornata* (16.8–21.1% d.w.)	*T. ornata* demonstrated a greater viscosity and yield than *S. mangarevense*. Both the species showed a high M:G ratio (1.25–1.42) compared to the reported literature. No seasonal variation was observed.	[31]
*Sargassum vulgare*	Formaldehyde-HCl-Na_2_CO_3_	16.9%	M/G ratio for *S. vulgaris* low density and *S. vulgaris* high density were higher than most Sargassum species ALG (1.56 and 1.27, respectively). Optimal conditions for extraction of ALG from *S. vulgaris* were 60 °C and 5 h duration. Newtonian activity seen for a solution fraction was 0.7% for SVLV, whereas, it was 0.5%. for the SVHV sample.	[32]
*Sargassum turbinarioides* Grunow	Formaldehyde-HCl-Na_2_CO_3_	10%	M/G ratio was 0.94, η < 1, M.W. (5.528 × 10^5^ g mol^−1^), and polydispersity index were low (1.43).	[33]
*Laminaria digitata* and *Ascophyllum nodosum*	Na_2_CO_3_ or NaOH after different acid pre-treatments (H_2_SO_4_ and HCl) at different temperatures	28.65 ± 0.92% to 78.02 ± 16.81%	Unrefined extracts produced films with appropriate mechanical characteristics without cationic complexation. The treatment with sodium carbonate resulted in extracts with good plasticizing capacity, while sodium hydroxide extraction guided to polymer chains with enhanced cross-linking ability. Ascophyllum films possessed radical scavenging property.	[34]
Tunisian seaweed (*Cystoseira barbata*)	high-temperature alkaline extraction	9.9%	M/G ratio was 0.59, pseudoplastic flow behavior. The emulsion formed was highly stable at acidic pH and less affected by temperature. CBSA exhibited DPPH radical scavenging activity (74% 33 inhibition at a concentration of 0.5 mg/mL). Excellent hydroxyl-radical scavenging activity, ferric reducing potential, and protection against DNA breakage were observed.	[35]
*Macrocystis pyrifera*	Ethanol route, HCl route, CaCl_2_ route	25–33%	Direct polymer precipitation with ethanol gave the best yield. The precipitation step with calcium and cation exchange gave an ALG with poor viscoelastic properties. A pH higher than 3.5 in the acid pre-treatment step amended the ethanol route, thereby preventing the ethanol linkages from being ruptured.	[36]
*Sargassum muticum*	conventional alkaline extraction and hydrothermal fractionation	5.04–10.09%	EC50 values for DPPH radical scavenging (0.72 and 1.18 g L^−1^ at 190 °C than at 220 °C, respectively) were comparable with synthetic antioxidants. However, at the minimum tested value (0.2 g L^−1^), the manufactured extracts at 190 °C appeared to be prooxidant. The AAC values that reached the maximum tested concentration at (0.5 g L^−1^) were similar to those for BHA and BHT.	[37]
*Sargassum* sp. (SRG) (genus *Sargassum), Turbinaria* sp. (TRB) (genus *Turbinaria*), *Hormophysa* sp. (RHT) (genus *Hormophysa*)	HCl-Na_2_CO_3_-EDTA	SRG 31 RHT 31 TRB 30	M/G ratio by ^1^H NMR 0.7–1.0, while after hydrolysis was 0.52–1.1. TRG with M/G <1 Gave a softer gel than SRG, while RHT, rich in mannuronic acid, gave the softest gel.	[38]
*Sargassum muticum*	Formaldehyde-HCl-Na_2_CO_3_	13.57 ± 0.13%	Optimum conditions for extraction are 86 °C temperature, 3% alkali, and 93% ethanol for 3 h. M/G was 1.08.	[39]

**Table 2:** Recent research on ALG-based formulations for improved actives delivery.

Devices	Model Drug/Drug	Composition	Preparation Technique	Delivery Site	Route of Administration	Key Features	References
Microparticles	Ropinirole hydrochloride	ALG	Spray-drying	Nasal epithelium	Intranasal	Excellent drug loading efficiency, in vitro rapid drug release (>95% in 30 min), negative zeta potential (−39.82 to and 70.07 mV), and no detrimental impact on the nasal mucosa.	[305]
Nanocomplexes	Doxorubicin and Temozolamide	Folic acid-CS-ALG	Complexation	-	-	Spherical diameter between 70–120 nm, Zeta potential ranging from 30–35 mV. In-vitro research on human cervical carcinoma cells and mouse fibroblasts showed more significant cytotoxicity or the dual-drug loaded formulation as compared to single-drug and free-drug formulations.	[306]
Nanoparticles	Insulin	CS-ALG	Self-assembly		Oral	The %EE of ALG-coated and CS-coated NPs were 81.5 ± 7.4% and 55.2 ± 7.0%, respectively. Effective plasma glucose reduction and prolonged insulin release after oral delivery to diabetic rats.	[307]
Microspheres	Retinoic acid	SA	One-pot method	eye	Intravitreal	Average particle size was 95.7 ± 9.6 μm. Stable and controlled release, no harm to the optic nerve, and physiological function assessed by VEP 5b and ERG b wave.	[308]
Microspheres	Clove essential oil	SA	Modified emulsification		Oral	% Yield of microspheres, loading capacity, encapsulation efficiency, and in vitro release were found to be 72.73%, 0.99 ± 0.3%, 24.77 ± 7.47%, and 48.64 ± 3.00% after 4 h, respectively. The microspheres showed a controlled in vitro release profile of the clove oil.	[309]
Nanoparticles	Amygdalin	CS-ALG	Ionic cross-linking		Mucosal	Zeta potential (−36 ± 0.88 and +32 ± 4.8 mV), mean size (around 119 nm), encapsulation efficiency (~90.3 ± 0.5%), effective swelling, and sustained release characteristics at pH 3.1, 5.0, 7.1, suitable mucoadhesive property in BioFlux system.	[310]
Multiple layer mucoadhesive films	Metformin	Thiolated SA and CMC sodium	Double casting followed by compression	Intrapocket	Mucosal	Homogeneous, thin, and strong films for effortless insertion into the periodontal cavity. Adequate mucoadhesion and sustained release for 12 h.	[311]
Nanoparticles	Naringenin	ALG coated CS	Dual crosslinking using Na_2_SO_4_ and CaCl_2_	Small intestine	Oral	Characterization by DLS, SEM, FTE, XRD, and pH-dependent dialysis study demonstrated excellent %EE of 91% and sustained flavonoid release. In vivo studies on rats showed significant anti-diabetic responses after oral delivery.	[312]
Nanoparticles	Miltefosine	ALG	Emulsification-external gelation method	mucosa	Oral	MFS-Alg NPs exhibited an average size of 279.1 ± 56.7 nm, polydispersity index of 0.42 ± 0.15, the zeta potential of −39.7 ± 5.2 mV, and %EE of 81.70 ± 6.64%. It presented no hemolysis or toxicity in G. mellonella larvae. Histopathological and CFU data show that MFS-Alg nanoparticles decreased the fungal load.	[313]
Microspheres	Ciprofloxacin	CS-coated konjac glucomannan/SA/graphene oxide	electrospinning	colon	Oral	The KGM/SA/GO microspheres were evaluated for their swelling rate (290%), drug loading (7.02%) and %EE (19.11%), zeta potential (−10.84, and −10.55 mV), and drug release (52% drug release after 20 h).	[314]
Nanoparticles	Curcumin diethyl disuccinate (CDD)	CS-ALG	Emulsification-ionotropic gelation	Human breast cancer	Oral	Encapsulated CDD in CANPs improved the stability and bio accessibility during the digestive stimulation and exhibited higher chemical stability when exposed to UV radiations. Bioavailability was enhanced five-fold. Greater cellular uptake and cytotoxicity in HepG2 cells than free CDD.	[315]
Nanocomposites	Doxorubicin	Fe3O4-SA-PVA-BSA	Co-precipitation/Ionotropic gelation	Cancer cells	Oral	The zeta potential ranged from −48.1 ± 2.3 to −22.4 ± 4.1 mV. The %EE varied between 36.2 ± 0.01 and 96.45 ± 2.12%. In vitro cytotoxicity tests using HepG2 and L02 cells demonstrated that DOX-loaded NPs (Fe3O4-SA-DOX-PVA-BSA) showed more significant cytotoxicity to HepG2 cells and non-toxic to L02 cells as compared to unloaded nanocomposites.	[316]

**Table 3:** Current approaches in ALG preparation for enhanced drug delivery.

**Devices**	**Model Drug/Drug**	**Composition**	**Preparation Method**	**Delivery Site**	**Applications**	**References**
Microspheres	Curcumin	ALG	Emulsion-gelation	Mucosa	Parenteral drug delivery	[317]
Layered double hydroxide Nanocomposites	Bovine serum albumin	ALG/CS	Ionic gelation	Intestine	Oral vaccine drug delivery	[318]
Multi-particulates	Dalfampridine	Tamarind seed gum/ALG	Ionotropic gelation	Intestine	Oral drug delivery	[319]
Microspheres	Icariin	CS/SA	Emulsification-internal gelation	Colon	Oral drug delivery	[320]
Nanoparticles	Doxycycline	CS/SA	Coacervation method	Spleen, blood	Oral drug delivery	[321]
Microbeads	Resveratrol	CS/ALG and ALG/sucrose	Ionotropic gelation	Intestine	Oral drug delivery	[322]
Microbeads	Chlorhexidine	CA	Internal gelation	Oral cavity	Periodontal drug delivery	[323]
Microspheres	Omega-3 rich oils (fish liver oil)	ALG/CS	Oil-in-water (o/w) emulsification, gelation, and microencapsulation	Intestine	Oral drug delivery	[324]
PECs/hydrogels	Bevacizumab	ALG	Dispersion	-	Local drug delivery	[325]
Nanoparticles	Lysozyme	Polymethacrylate/ALG	Coacervation	-	Delivery system	[326]
Hydrogels	Deferoxamine	CS/ALG with poly(d,l-lactide-co-glycolide)	Mixing	Intestine	Oral drug delivery	[327]
Hydrogels	5-Fluorouracil	Succinoglycan dialdehyde cross-linked hydrazine functionalized ALG	Ionic cross-linking		pH-controlled drug delivery	[328]
Films	Omeprazole	Hydroxypropyl methyl cellulose (HPMC)/Methyl cellulose (MC)/SA/carrageenan(CA)/metolose(MET)	Film casting	Stomach	Buccal drug delivery	[329]
Floating In situ gel	Celecoxib	SA/PEG	Ionic cross-linking	Site of inflammation/edema	Oral sustained drug delivery	[330]
Hydrogel beads	Ibuprofen	ALG-magnetic nitrocellulose (m-CNC)	Ionic cross-linking	-	Drug delivery	[331]
Hydrogel encapsulated microspheres	5-Fluorouracil	CS/ALG/gelatin	Emulsion cross-linking	-	Drug delivery	[332]
Microspheres entrapped hydrogels	Methotrexate, loaded Calcium Carbonate, and Aspirin	ALG and sodium CMC crosslinked with Ca^2+^ ions	Co-precipitation	-	Drug delivery	[333]
Films	Tamoxifen	ALG/CS	Spray-assisted LbL technique	-	Drug delivery	[334]

## Data Availability

Not applicable.

## Abbreviations

5-FU	5-Fluorouracil
AAC	antioxidant activity coefficient
Aah	Androctonus australis hector
AGM	agomelatine
AGMPs	sodium alginate(AG)based microparticles
ALG	Alginate
Alg-APBA	3-aminophenylboronic acid-modified alginate
ALG-CNC NPs	Alginate cellulose nanocrystals nanoparticles
AmpB	Amphotericin B
ARPE-18	Human retinal pigmented epithelium cells
ATO	Arsenic trioxide
BALB/c	Bagg Albino mouse strain
BBSA	Bifurcaria bifurcata
BCG	Bacille Calmette–Guérin
BET	Brunauer–Emmett–Teller
BSA	Bovine serum albumin
CA	Calcium alginate
CAC	composite alginate collagen
CaG	calcium gluconate
CANPs	chitosan/alginate nanoparticles
CBSA	C. barbata sodium alginate
CD	Crohn’s disease
CDD	Curcumin diethyl disuccinate
CFX	Cefixime
CMC	carboxymethyl cellulose
CNCs	Cellulose nanocrystals
COPD	Chronic obstructive pulmonary disease
CpG ODN	CpG oligodeoxynucleotides
CS	Chitosan
CTFR	cystic fibrosis transmembrane conductance regulator
DDS	Drug delivery system
DLS	Dynamic light scattering
Dox	Doxorubicin
DPPH	1,1-Diphenyl-2 picrylhydrazyl
DTZ	diltiazem
ECT	Encapsulated-cell therapy
EE	Entrapment efficiency
ERG	electroretinogram
F–ERG	Flash electroretinogram
Fe_3_O_4_-SA-DOX	Fe_3_O_4_-sodium alginate–doxorubicin
FITC	fluorescein isothiocyanate
FSSA	*Fucus spiralis* L.
G	4-α-L-guluronic acid
GA	gallic acid
GDNF	glial-derived neurotropic factor
GL	glycyrrhizin
GO	graphene oxide
GSM	glyceryl monostearate
HA	Hyaluronic acid
HaCat	Cultured Human Keratinocyte
HAI	Haemagglutinin inhibition
HAp	hydroxyapatite
HB-CNPs	HbsAg-loaded chitosan nanoparticles
HbsAg	Hepatitis B surface antigen
HEK293	Human Embryonic Kidney cells
HEMA	(Hydroxyethyl)methacrylate
HPBCD	Hydroxypropyl beta cyclodextrin
HPMC	Hydroxypropyl methylcellulose
HP-β-CD	hydroxypropyl-beta-cyclodextrin
i.m.	intramuscular
IL	Interleukins
KF	ketotifen fumarate
KGM	Konjac glucomannan
M	1,4-linked-β-D-mannuronic acid
MA	Methyl acrylate
MAPTAC	Methacryolyl aminopropyl trimethyl ammonium chloride
m-CNC	magnetic cellulose nanocrystal
MFS	Miltefosine
MIC	Minimum inhibitory concentration
MMP-2	Matrix metalloproteinase-2
M-MSNs	magnetic mesoporous silica nanoparticles
MNs	Microneedles
MPs	Microparticles
MTT	3-(4,5-dimethylthiazol-2-yl)-2,5-diphenyl tetrazolium bromide
NLC	nanostructured lipid carriers
nMBA	N, N′-methylenebisacrylamide
OA	Oxidized alginate
PAH	poly(allylamine hydrochloride)
PBMC	Peripheral blood mononuclear cell
PCL	Polycaprolactone
PECs	polyelectrolyte complexes
PEG	Polyethylene glycol
PEG-co-PCL	poly (ethylene glycol)-co-poly(-caprolactone)
PEG@VTMSg-CS	Polyethylene glycol coated vinyl trimethoxy silane-g-chitosan
PEM	polyelectrolyte multilayers
PF	Pluronic
PFD	pirfenidone
P-gp shRNA	P-glycoprotein short hairpin RNA
PGX	Pressurized gas expanded liquid
PLC/PEG	poly(caprolactone)/polyethylene glycol
PLGA	poly (lactic-co-glycolic acid)
PLL	Polylysine
PMX	Polymyxin B
PR8-ALG	PR8 influenza virus sodium alginate
PR8-CHT	PR8 influenza virus chitosan
PR8-TMC-ALG	PR8 influenza virus sodium alginate-coated chitosan and trimethyl chitosan
PSSCMA	poly(4-styrenesulfonic acid-co-maleic acid) sodium salt
RBA	Relative Bioavailability
RCS	Royal College of Surgeons
RPA	Relative Pharmacologic Availability
s.c.	subcutaneous
SA	sodium alginate
SA-ATO-NPs	make ATO-loaded sodium alginate nanoparticles
SCC	squamous cell carcinoma
SD rat	Sprague Dawley rat
SGF	simulated gastric fluid
SH	sulfhydryl
SpBMP-9	Small peptide Bone Morphogenetic Protein 9
SRG	*Sargassum* sp
SS	salbutamol sulfate
SWCNT-GI	single-walled carbon nanotube modified by glucose
TA	Tannic acid
TCS	thiolated-chitosan coated sodium alginate
TEM	Transmission electron microscopy
THSG	2,3,5,40 -tetrahydroxystilbene 2-O-β-D-glucoside
TRB	*Turbinaria* sp
TSA	thiolated sodium alginate
UTI	Urinary tract infection
VEP	visual evoked potential
VLF-AG-NPs	venlafaxine alginate nanoparticles
XRD	X-ray Diffraction
